# Management Platforms and Protocols for Internet of Things: A Survey

**DOI:** 10.3390/s19030676

**Published:** 2019-02-07

**Authors:** Jonathan de C. Silva, Joel J. P. C. Rodrigues, Jalal Al-Muhtadi, Ricardo A. L. Rabêlo, Vasco Furtado

**Affiliations:** 1 National Institute of Telecommunications (Inatel), Santa Rita do Sapucaí MG 37540-000, Brazil; jonathancs@inatel.br; 2 Instituto de Telecomunicações, 1049-001 Lisboa, Portugal; 3 College of Computer and Information Sciences, King Saud University, Riyadh 11653, Saudi Arabia; jalal@ccis.edu.sa; 4 Center of Excellence in Information Assurance, King Saud University, Riyadh 11653, Saudi Arabia; 5 Department of Computing (DC), Graduate Program in Computer Science (PPGCC), Federal University of Piaui (UFPI), Ministro Petronio Portela Campus, Teresina 64049-550, Piauí, Brazil; ricardoalr@ufpi.edu.br; 6 Graduate Program in Applied Informatics, University of Fortaleza (UNIFOR), Fortaleza CE 60811-905, Brazil; vasco@unifor.br

**Keywords:** Internet of Things, IoT management, network management, device management, management platform, protocols

## Abstract

Internet of Things (IoT) management systems require scalability, standardized communication, and context-awareness to achieve the management of connected devices with security and accuracy in real environments. Interoperability and heterogeneity between hardware and application layers are also critical issues. To attend to the network requirements and different functionalities, a dynamic and context-sensitive configuration management system is required. Thus, reference architectures (RAs) represent a basic architecture and the definition of key characteristics for the construction of IoT environments. Therefore, choosing the best technologies of the IoT management platforms and protocols through comparison and evaluation is a hard task, since they are difficult to compare due to their lack of standardization. However, in the literature, there are no management platforms focused on addressing all IoT issues. For this purpose, this paper surveys the available policies and solutions for IoT Network Management and devices. Among the available technologies, an evaluation was performed using features such as heterogeneity, scalability, supported technologies, and security. Based on this evaluation, the most promising technologies were chosen for a detailed performance evaluation study (through simulation and deployment in real environments). In terms of contributions, these protocols and platforms were studied in detail, the main features of each approach are highlighted and discussed, open research issues are identified as well as the lessons learned on the topic.

## 1. Introduction

Initially, computer networks were created for communicating as a mean of sharing endpoint devices with the same standards of networks, protocols, and operating systems. However, the fast evolution of networks combined with a reduction of computational resources costs motivated the increase of computer networks in all markets [1]. Considering this scenario, it becomes increasingly necessary to manage the network environment to keep it working properly. Network management is required to maintain the entire network structure working, thus meeting the user needs and the administrators’ expectations.

Due to the emergence of the Internet of Things (IoT), it is expected an exponential growth of network endpoint devices (NEDs) becoming a challenge in the areas of infrastructure, security, energy saving, among others [2]. The continued growth in the number and diversity of network components has also contributed to the fact that network management activity has become increasingly indispensable [3]. The benefits of integrating a company’s computing systems of different nature and sizes as a way of distributing tasks and sharing available resources are now a reality. For this reason, an efficient data management system is required by IoT networks, so the information is always available wherever and whenever requested [4].

IoT management presents two main scopes: devices and networks. In each of them, there is a huge variety of protocols and management platforms to minimize the challenges (presented later) [5]. Given the number of existing protocols and platforms, an evaluation should determine which IoT management protocols are capable of efficiently satisfying the application requirements and which platforms support these protocols for real environments (real deployment).

**Figure 1:**
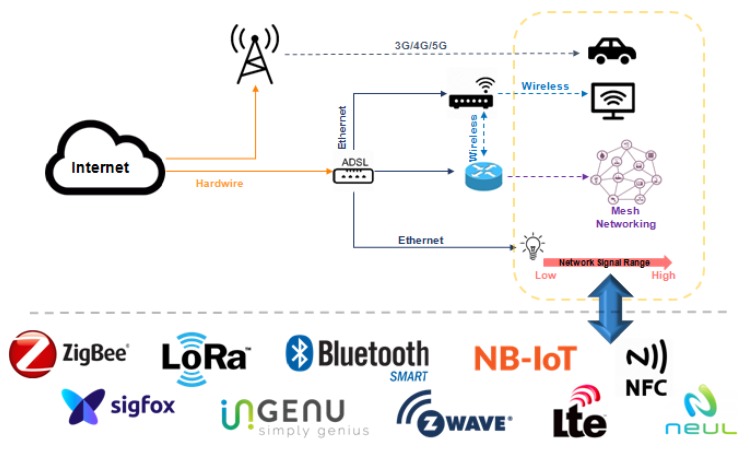
Illustration of an IoT Network Architecture and a plethora of available protocols.

Communication between devices performing machine-to-machine communication (M2M), Wireless Sensor Networks (WSNs) for monitoring and control processes, and the interconnection of WSNs with the Internet are examples of some challenges of managing an IoT network [6]. Network devices using software-based communication (known as software-defined networks—SDN) gathers, detects, and configures data from sensors, thus creating the context of managing a network. New technological approaches focusing on IoT are emerging, as Fog/Cloud technologies [7], and they are compatible with constrained portable devices and with old management protocols, therefore being an IoT trending topic. Figure 1 presents a typical scenario involving different communication technologies and a gateway where connected devices collect information from the environment (e.g., temperature, luminosity, movement, etc.) and report data to an IoT management network entity.

One of the most important challenges in this IoT scenario is the network device heterogeneity [8,9]. Devices can support different communication protocols with different formats and data types, memory and processing capacity [10]. Another important factor is the data set produced in real time and the implicit semantics imposing challenges regarding the configuration and infrastructure of IoT environments [11]. An illustration of the heterogeneity of protocols for IoT is shown in Figure 1.

The complexity of IoT Network Management compared with traditional Transport Control Protocol (TCP)/IP networks management is also greater than WSNs [12]. IoT needs to support networking devices and services that involve (i) the use of a plethora of devices with diverse characteristics, and (ii) the IoT networks devices interaction through local or remote management context awareness. WSNs should manage frequent communications failures and low security of wireless links (i.e., the MANNA architecture [13]), and this management must also be context aware. The available IoT management architectures partially attend to these features [14]. Therefore, this survey elaborates on a deep study of the related literature focusing on available solutions, tools, and policies, including approaches for IoT networks and end devices management. Among the available technologies for IoT, an evaluation was performed using features its heterogeneity, scalability, supported technologies and security. Based on this evaluation, the most promising technologies were chosen for a detailed performance evaluation study (through simulation and deployment in real environments). Then, the main contributions of this paper can be summarized as follows:

- An extensive review of the related literature considering network management protocols and platforms for IoT;
- Requirements analyses for IoT network and devices management;
- A comparative analysis of the available IoT protocols and network management platforms for IoT;
- Identification of challenges and open research issues on IoT management and its importance for further studies on the topic.

The paper is organized as follows. Section 2 addresses important background information on network and device management. In Section 3, IoT Network Management and its requirements are introduced. The IoT Network Management protocols and the most relevant platforms available in literature are detailed in Section 4 and Section 5 respectively. IoT Device Management and its requirements are introduced in Section 6. Then, the IoT Device Management protocols and platform technologies are studied in Section 7 and Section 8. Section 9 presents a performance evaluation study of IoT management technologies and proposes open research issues based on obtained results. Finally, the lessons learned and the main conclusions are addressed in Section 10.

## 2. Background

Computer networks are composed by heterogeneous communication devices and sharing resources [15]. Computer networks management emerged after a rapid evolution of network technologies, in addition to a major effort to reduce the costs of computing resources [16]. The offered services range from simple resources sharing to current technology and assume that every object can be connected to the Internet. This is known to as the IoT. Network management goals controlling and monitoring network elements, physical or logical, ensuring a certain quality of service (QoS) level. To accomplish this task, define network management as a collection of tools for monitoring or managing devices [17]. The traditional network management model can be summarized as follows: (i) data collection from monitoring managed resources automatically, (ii) diagnosis to analyze and solve identified problems throughout the monitored data, and (iii) action or control to solve a problem or modify the state of a device [18].

For Kurose et al. [18], a network without management mechanisms can present problems such as interference in data traffic, lack of data integrity, high congestion rates, resources that can be misused or overloaded, as well as security problems. According to Gabdurahmanov [19], network management can be difficult for three reasons: (i) the managed network is heterogeneous because it contains hardware and software components manufactured by various companies; (ii) technology can change continuously with new services available; and (iii) the managed networks are large and the network nodes may be distant from other nodes.

**Figure 2:**
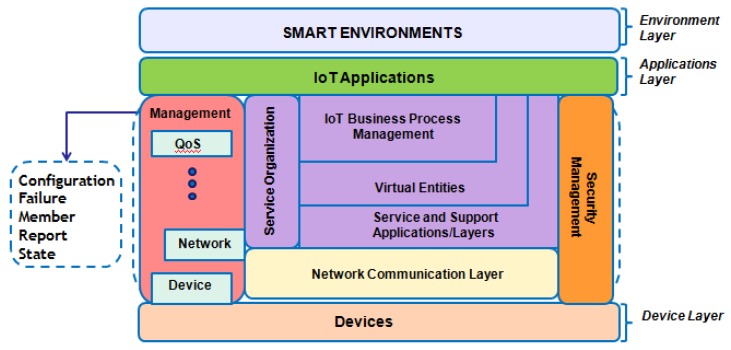
Internet of Things Reference Architecture (IoT-RA).

Kurose et al. [18] states that ISO has created an IoT reference architecture (RA) in which network management includes five functional areas, as follows: performance management, failure, configuration, accounting, and security (as shown in Figure 2). Performance management intends to analyze, measure, inform, and control the performance of different network components, i.e., routers and hosts. In failure, the purpose is to log, detect, and react to network failure conditions. The division between fault management and performance management is undefined [20]. Failure management can occur through transient network failures, such as the interruption of service on a link or routing software. Performance management, however, takes a long-term approach.

The administrator can know the managed network devices and their hardware configurations through management actions [21]. Accounting is intended to allow the network administrator to specify, register, and control access to users and devices of the network. Quotas and usage charges for privileged access to the network resources make up the accounting management. According to organization policies defined, the network resources access is performed with security management.

**Figure 3:**
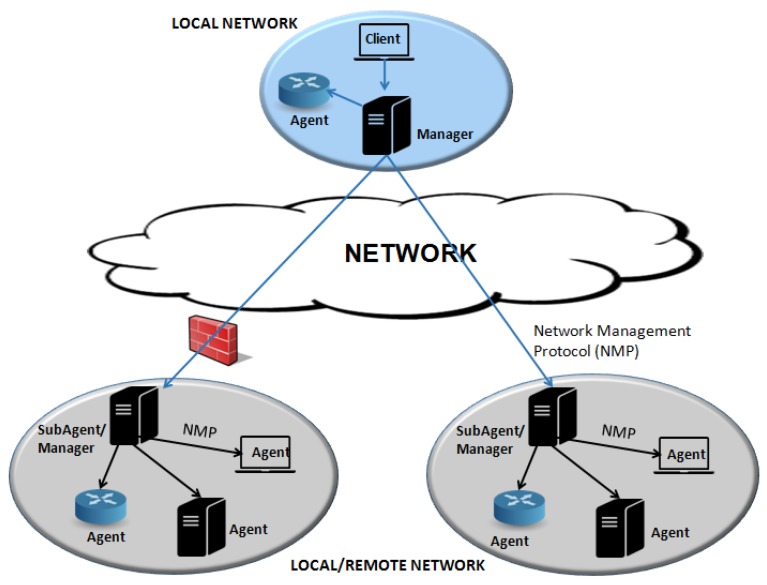
Components of network management systems.

In Figure 3, the general architecture of network management systems presents the following four basic components: elements, stations, protocols, and information on network management [18]. They are briefly described below.

**Agent**: The managed elements have a software that allows monitoring and controlling the equipment through one or more management stations;

**Manager**: Management station communicates with the agents, either for monitoring or controlling them. Usually, the management station offers an interface through which authorized users can manage the network;

**Protocol**: The standard protocol, used for operations of monitoring (reading) and control (writing); it is necessary for information exchange between manager and agent devices;

**Management information**: The management information has the data that can be referenced for operations by the management protocol, i.e., the managers and agents can exchange data to obtain information such as the Simple Network Management Protocol (SNMP) protocol [22].

Since the proposal of IoT years ago, many ideas have had three main constraints that restrict its development [23]: (i) proprietary communication protocols, (ii) security and privacy, and (iii) inconvenience to manage. Thus, the paper focuses on network and Device Management and if it is feasible to address management constraints.

Due to the specific characteristics and challenges of IoT, devices cannot be managed using only the traditional management tools. Thus, IoT management has two categories: IoT Network Management and IoT Device Management. IoT Network Management is required to collection and analysis large volumes of data from IoT platforms and, consequently, provides efficient decisions and/or actions. IoT Device Management is required to provide the device location and status information, e.g., update embedded software, disconnect some stolen or unrecognized device, modify security and hardware configurations, locate a lost device, and even enable interaction between devices.

## 3. IoT Network Management

IoT Network Management often needs to adapt the unknown topology of these networks by providing device location and status information. Managing services should have the capacity to disconnect and locate lost devices, modify security settings, delete device data, and more. Delicato et al. [24] states that management should consider the possibility of integrating and using the devices not previously planned in the environment opportunistically.

**Figure 4:**
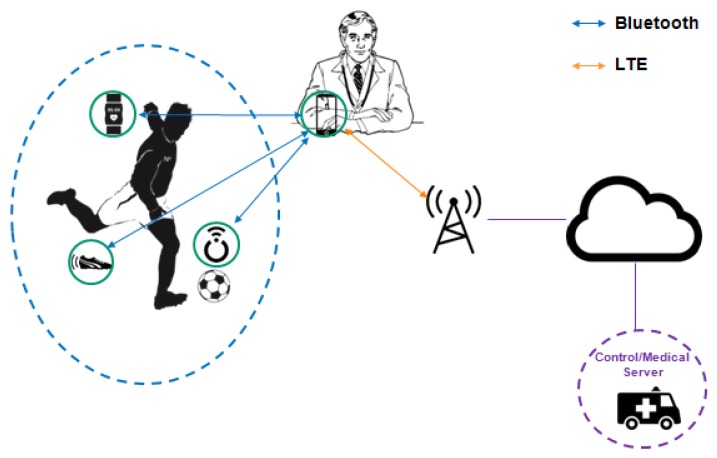
Illustration of an IoT network environment.

An IoT network environment can have various connected devices in the same network, such as a health sensor, a control/medical server, a Web report of statistics and a smartphone, as illustrated in Figure 4. Thus, it is important that a management platform enables the devices to dynamically detect other devices present in the environment to meet the requirements of the applications.

The main difference between WSNs, AdHoc networks (MANETs), and IoT networks is the characteristic of heterogeneous devices and topologies performed by these networks [25]. All the time, new devices compose these networks, therefore, the IoT management platforms require a customized module (driver) to translate device functionality into the platform. Some platforms for WSN and AdHoc networks can be used to IoT networks.

### 3.1. Requirements

IoT management solutions must meet some requirements [14]. For example, there must be interoperability between platforms and network devices. The platform must obtain the connected devices dynamically through discovery and management. A solution should be context aware and support scalability (considering indications of intensive usage). Security and dynamic adaptation should keep data integrity and privacy guaranteeing devices availability and QoS.

Existing IoT management platforms, however, only partially meet these requirements [14]. Due to lack of standardization, IoT management needs to specify data and information models, in which these models are used to define a format for storing and deploying other management services. In addition to the above requirements and other aspects such as security, authentication and authorization, there are characteristics in the scope of local and global management which are discussed in [26].

### 3.2. Features

In the IoT reference architecture [27], network management includes functional components. Configuration (self-configuration) is responsible for initializing system configuration, i.e., collect and store the device configurations, tracking configuration changes, and planning future extensions.

The self-aware component, Failure, identifies, isolates, recovers, and records failures in an IoT system. For each occurrence of a failure, a notification is sent to the Failure component with the objective of collecting more data to identify the type and degree of the problem.

Member is responsible for monitoring and recovering members. This component allows recovering members of the system while obeying a certain filter and allows the subscription to receive updates of register/unregister member metadata in the database.

Report refines and maintains the history of the information provided by management devices, e.g., to determine the efficiency of the current system through the collection and analysis of performance data.

State goals self-monitoring of the IoT system with the past, present, and future devices states. It is required by the Failure component, having the functions of changing or applying a particular state in the system. It also checks the consistency of commands provided for this function and monitors the state, which makes it possible to predict and update the state for a certain time or to recover the state of the system through a history.

## 4. IoT Network Management Protocols

The section elaborates on the most relevant IoT Network Management protocols.

### 4.1. Simple Network Management Protocol (SNMP)

The objective of the SNMP is to find and fix the bugs or problems of a network [28]. Through SNMP agents, the network administrator can view network traffic statistics and is able to change its configurations after analyzing this data. Defined at the application level and standardized by the Internet Engineering Task-Force (IETF) [29], the SNMP uses the User Datagram Protocol (UDP) transport protocol to send messages over the network without delivery guarantee.

Over the last years, other protocols have used the same concept, e.g., NETCONF was created to replace SNMP. The SNMP continues to dominate the network management market, mainly because of its simplicity of implementation, since it consumes fewer network resources and processing, which allows the inclusion of very simple equipment. According to SNMP Research International et al. [30], there was an incremental development of three versions of the SNMP, as described below:

- SNMPv1: it offers a management solution with low cost and simple implementation, but with a lack of authentication and security mechanisms and limitation in the performance of messages in very large networks.
- SNMPv2c: it created the management of decentralized networks, allowing the existence of more than one management station and, consequently, the exchange of information between them. In SNMPv2, it was not possible to reach a consensus regarding the security standard to be used in SNMP, and there was an addition of other request types such as get-request, get-next-request, set-request, response, and snmpV2-trap.
- SNMPv3: it published a set of documents defining a framework to incorporate security features into full capacity (with the SNMPv1 or SNMPv2 features). The SNMP architectural model includes a Management Server and network devices. Servers monitoring and control the network devices with events. The network devices include equipment as hosts, gateways, terminal servers, that have managing agents used to be handled by a server. The SNMP protocol is used to exchange data between a server and network devices.

**Figure 5:**
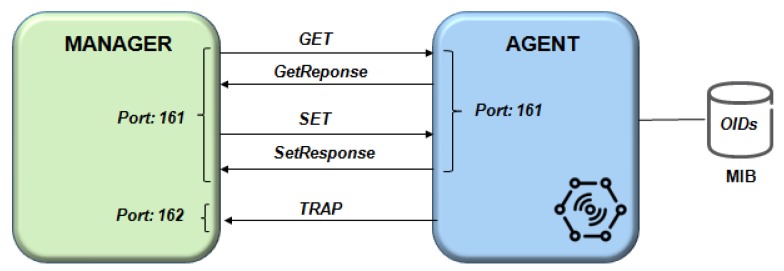
Illustration of the SNMP Protocol Architecture.

Figure 5, shows some of the possible interactions between manager and agent through the SNMP protocol [31].

An SNMP device can be connected to other devices, performing machine-to-machine communication [32].

The SNMP Agent is a software installed in a device to support network management. It answers the queries from SNMP managers and sends a trap message to some events (according to their priority). The Management Information Base (MIB) is a virtual database with object identifiers (OIDs) organized in a tree structure to keep information about Device Management in a communication network.

The ASN.1 (Abstract Syntax Notation One) notation [33] is a language developed by ITU-T and chosen by ISO for the definition of the MIB manageable objects. It uses object-oriented concepts to define a resource, so that its attributes can be performed by this resource, when applicable.

**Figure 6:**
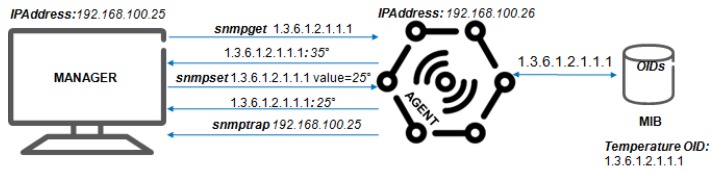
Illustration of the SNMP protocol for IoT devices.

The SNMP is a non-connection-oriented protocol does not require a prior or subsequent action to send messages. Thus, the protocol messages will no guarantee the destination is reached. It is a simple and robust protocol, yet powerful enough to solve the difficult problems presented by managing heterogeneous networks such as an IoT network, as shown in Figure 6. Therefore, the key problems to manage the sensors in IoT involves the MIB design and the development of manager and agent software.

*Pros: Simple and easy protocol to be developed.*

*Cons: No network configuration resources.*

#### Use Case

The SNMP protocol is a base unit that provides a centralized platform for operations and facility management teams to monitor sensor conditions and configure threshold-based alerts. It aims to monitor and collect data from environmental sensors and to integrate data from equipment such as generators and other devices enabled to integrate with intelligent sensors through SNMP, as demonstrated by OpenNMS, which is an open-source platform [34].

### 4.2. Network Configuration Protocol (NETCONF)

**Figure 7:**
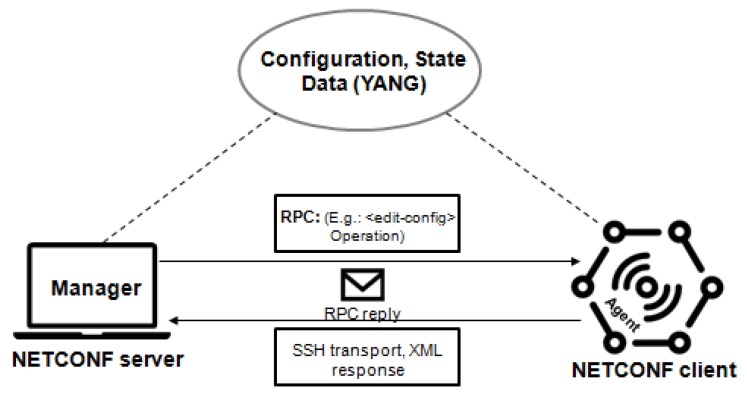
Illustration of the NETCONF Protocol for IoT Architecture.

The NETCONF is a protocol for network configuration and monitoring, as defined by the IETF [35] and, therefore, has better features than the SNMP, which had as its weakest point the absence of network configuration resources, i.e., the interface BER (Basic Encoding Rules) and proprietary MIBs. The NETCONF protocol was developed to be the natural successor to SNMP, because SNMP is focused on monitoring and not on network configuration while NETCONF uses mechanisms that allow the installation, manipulation, and removal of network device configuration through a client-server implementation, as shown in Figure 7.

After establishing the secure transport session between client and server, the NETCONF protocol sends a *HELLO* message to announce the protocol capabilities and supported data models. NETCONF also supports the subscription and receipt of event notifications asynchronously as well as the partial closing of a current configuration of a network device. This feature allows multiple editing sessions, streamlining the configuration process. NETCONF allows monitoring and management of an autonomous entity (the NETCONF manager) that uses the repository of data, sessions, closings, and statistics available on the NETCONF server.

The NETCONF protocol transports this information to an application manager, who can infer the required settings for the network devices. YANG is a formal language with clear text of the data model with syntax and semantics that allow the construction of network applications [36]. The YANG model can be translated into an XML (Extensible Markup Language) or JSON (JavaScript Object Notation) file, structured in a tree for each module, with properties that correspond to the functionalities of the device and declarations of types, data, constraints, and additions of reusable structures.

Heterogeneous networks are characteristic of the IoT, and the NETCONF protocol is used to efficiently manage and resolve issues of this network. Most operating systems developed for IoT [37] such as TinyOS and Contiki OS already have the NETCONF protocol built into their operating system.

*Pros: Robust and security features.*

*Cons: a data model and an architecture of standardized implementation.*

#### Use Case

Currently, most vendors, e.g., Cisco, already use the NETCONF protocol on their equipment as a standard model. Another example, the OpenFlow devices controller communicates with connected devices in an SDN architecture, also defining a protocol for such communication. The OpenFlow provides means to control network devices using NETCONF, without the need for manufacturers to expose the code of their legacy products [38].

### 4.3. Open vSwitch Database (OVSDB)

Open vSwitch Database (OVSDB) is a management protocol in a SDN environment [39]. Most network devices allow remote configuration using legacy protocols, such as SNMP. The goal of Open vSwitch (OVS) consists in creating a modern programmatic management protocol interface—OVSDB.

**Figure 8:**
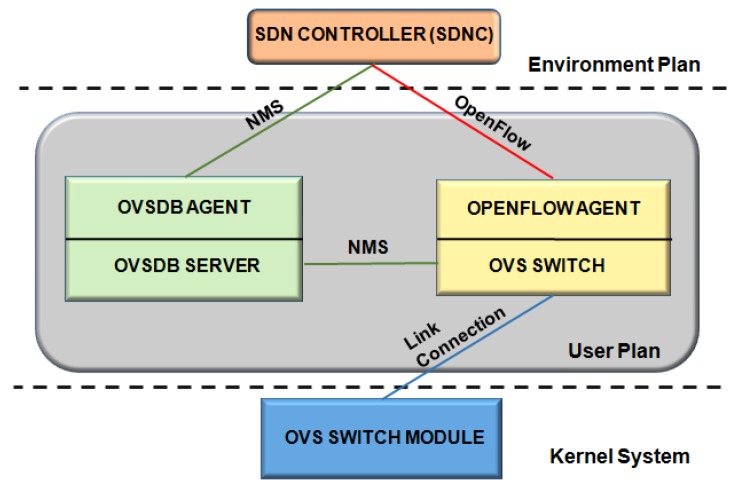
Illustration OVSDB Protocol for IoT Architecture.

According to Figure 8, the OVSDB management protocol handles OVS that consists of a database server (OVSDB Server), a virtualized switch (OVS Switch) and, optionally, a module for fast-path forwarding. Each OVS is managed by, at least, one manager. An OVS module supports several data paths referred to as “bridges”, where this controller uses OpenFlow.

The OVSDB protocol interface to execute the configuration and management operations on the OVS instance. OVSDB is used to create/delete/modify bridges, ports, and interfaces. The OVS represents an evolution of network management protocols, allowing programming and configuring bridges, ports, and interfaces for SDN equipment platforms and Network Functions Virtualization (NFV) [40].

*Pros: Interoperability of the networks and SDN management network.*

*Cons: No standard model to other networks and security features.*

#### Use Case

The open-source OpenDayLight platform for SDN uses open network management protocols, i.e., SNMP, OVSDB, and NETCONF to provide modular functions, extensible control, and network device monitoring [41].

### 4.4. Internet of Things Platform’s Infrastructure for Configurations (IoT-PIC)

**Figure 9:**
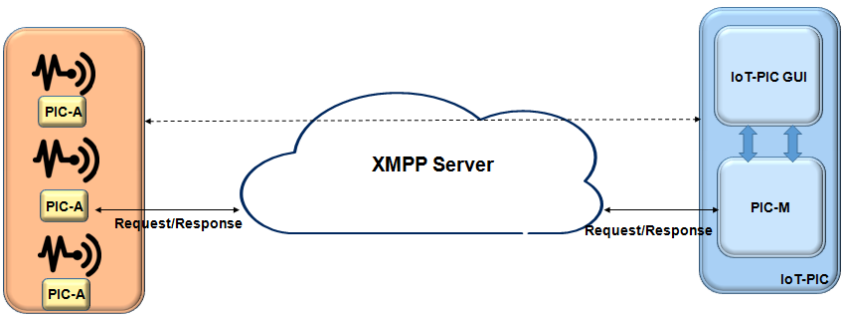
Illustration of the IoT-PIC architecture.

The IoT-PIC allows network management to perform the platform commissioning installed in the network. The IoT-PIC architecture is similar to the SNMP protocol previously described, as shown in Figure 9. It has the possibility of performing any configuration or composition of hardware and software resources.

The IoT-PIC architecture have two levels, the global and local, and it is composed by two components, in which the communication among these components is made through the Extensible Messaging and Presence Protocol (XMPP) protocol [42]: an IoT-PIC Manager (PIC-M) at a global level and an IoT-PIC Agent (PIC-A) at a local level.

The XMPP protocol is an open-source IETF standard protocol based on XML for network management in IoT contexts, which allows real-time messaging, the information exchange and request/response services. The XMPP performance of latency, scalability, and robustness has been widely demonstrated during the years [43].

The PIC-M module is used to manage the configuration and access of the other modules to the platform. It interacts as an interface to applications and other platform components.

The PIC-M functionalities consist in notifying the applications on the status of the device available in the middle-ware, requesting configuration information from the PIC-A via “get” and “set”, through an XMPP command, and updating the configuration of devices through PIC-A via XMPP.

The IoT-PIC uses the publish-subscribe, in which subscribers only receive messages of interest, without information on the publishers, which allows the complete decoupling of the devices. Each platform is associated with a PIC-A and responds to the management of the PIC-M device. The configuration and interconnection of devices are assigned to the PIC-A, e.g., adding and removing the connection.

The IoT-PIC deploys the discovery functionality of devices through the XMPP protocol. New devices connected are automatically registered to the network, describing their functionalities with a common format [44]. Particularly, in the proposed solution, when a new device connects to the network, the manager of this network publish-subscribe joins in PIC-M for all discovered resources.

A resource example is a sensor that measures humidity and temperature. Context Manager can create location-related nodes where devices can enter their location allowing navigation of the tree from the root. First, the PIC-M creates a collection node with a given device id, containing two nodes, in which the first node has the temperature function and the second node has the humidity function. The resources of the nodes used in the service discovered by PIC-M are associated with the resource types list, i.e., *Humidity Sensor* and *Temperature Sensor*. The functions of the nodes are associated with the operation list, i.e., *getTemperature* and *getHumididity*. This list creates the entire hierarchy of nodes and, if the user does not indicate the parameter, the entire list is returned.

*Pros: Interoperability and global/local management.*

*Cons: No mature protocol and security feature.*

#### Use Case

The IoT-PIC is used in an energy efficiency scenario. The IMPReSS platform [45] includes energy saving and alarm system applications to allow sensor, lights, and smart plugs into the platform. To save energy, the Energy Saver manages the light through the PIC-M and tells the PIC-A of the lights management component to publish/subscribe node of the device in order to receive its events; e.g., in a classroom, detects motion sensor if a row of seats is empty, in which case the lights are automatically switched off.

The GUI interface converts the XML returned by response of the PIC-M into a user-friendly form. This platform allows integrating new devices without need modifications to the deployment environment.

### 4.5. IEEE 1905

IoT environments depend on several media access control (MAC) protocols. The challenge of interoperability between technologies needs to be discussed. IEEE 1905 is a standard focused on the convergence of digital home network and offers an abstraction layer to all these heterogeneous MAC protocols.

**Figure 10:**
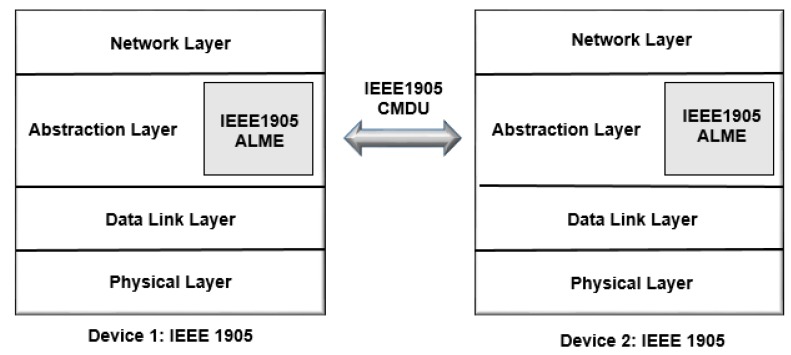
Illustration of the protocol structure for IEEE 1905.

The goal of IEEE 1905 is to define a common standard that establishes home network technologies for a data and control service access point. Each interface can transmit and receive packets, regardless of underlying technologies or layers, as shown in Figure 10 [46].

**Figure 11:**
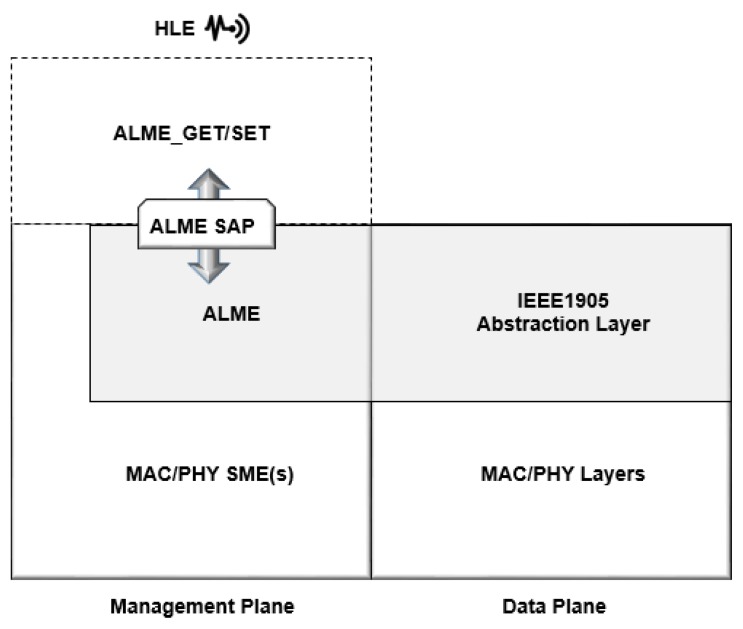
IEEE 1905 standard network management architecture.

An intermediate layer used to exchange messages (Table 1), is called Control Message Data Units (CMDUs), with all standards-compatible devices. In Figure 11, all the IEEE 1905 deployed devices with Abstraction Layer Management Entity (ALME) protocol have neighbor discovery, topology exchange and rules, measured traffic, and security associations following the layers presented.

The protocol introduces an intermediate abstraction layer to the logical link control (LLC) and one or multiple MAC. The service access points (SAPs) holds many networking technologies, to support advanced network management features such as auto-configuration, QoS, path selection and discovery. This layer simplifies setup, e.g., by eliminating the need for a user to enter different passwords to access each link [47].

This ALME SAP entity can provide management services to MAC, physical layer (PHY) and higher layer entities (HLEs) [48]. It also provides advanced network management features including discovery and interface selection.

*Pros: Interoperability and common standard model to devices.*

*Cons: No mature protocol and security feature.*

#### Use Case

nVoy is an IEEE 1905 standard program [49] that provides the services to maximize and simplify the overall performance of a home network. The reliability is provided through the abstraction layer to established power line, wireless, coaxial cable, and Ethernet home networking technologies—IEEE 1901/HomePlug® AV, Wi-Fi, MoCA®, and Ethernet, allowing to provide common setup procedures for establishing connected devices, secure links, and network management.

### 4.6. LoWPAN Network Management Protocol (LNMP)

The LNMP is a management architecture suitable for 6LoWPAN networks [50]. With LNMP architecture is possible to reduce the cost communication and, therefore, increasing the lifespan of the network. The LNMP main characteristic is to allow interoperability with SNMP. In terms of communication and complexity, the SNMP is considered impracticable due to the limited device’s resources.

**Figure 12:**
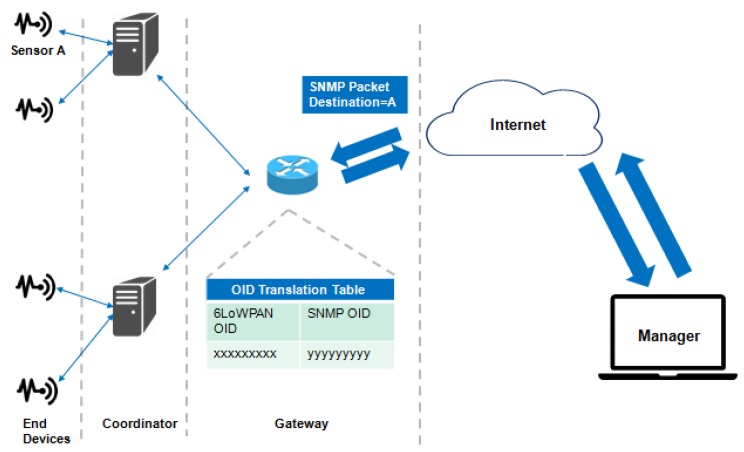
Illustration device level monitoring procedure for LNMP Protocol.

This architecture (Figure 12) allows the discovery of devices in a network with help of the coordinators in the monitoring and management. The SNMP is an application layer adapted protocol to run over IPv6, so uses this protocol to the adaptation layer 6LoWPAN [51]. The popular solution NET-SNMP [52] includes the adapted IPv4 and IPv6 for IoT network.

Exists two successive management operations that entities within the 6LoWPAN performed. First, Network Discovery is executed to monitor the device state in the architecture. The second step, after discovered devices, is the management of available devices.

To discover “live” devices manually, intense use of the resources is needed, and thus, the Network Discovery is a procedure created for an automated network state discovery, which is necessary given the WSN characteristics for their continued deployment. In this proposal [53], the Network Discovery uses the automated monitoring of the network state distributed by a 6LoWPAN network. The coordinators responsible to maintain the information about device state and reporting of subordinate devices has the device discovery feature. Bandwidth is a scarce resource in a sensor network and this feature reduces communication costs. The sensing and processing are usually lower than the communication cost.

It is desirable to monitor the device’s status within the 6LoWPAN in a standard management protocol, e.g., SNMP protocols. However, the bandwidth available is a factor limited for application layer payload [54]. Therefore, SNMP is inviable due complexity of transport and communication into 6LoWPAN networks. Nonetheless, the reuse of network protocols is a goal of the 6LoWPAN, especially because of the interoperability of devices with SNMP. The SNMP message is translated to a UDP-based query when arrives from an NMS. It contains identifiers objects that are retrieved by the Device Agent. Likewise, these objects are translated to SNMP format when arriving at the gateway.

The data validity is the most important consideration to management architecture. The performance of the network management can be calculated with query-response delay and the increasing number of nodes. Likewise, another way is analysis the computation overhead with query load. The reliability introduces a delay of 25 ms to a query and reaches up 50%. Queries with five hops proposed a delay of 100 ms or more gave 100% reliability.

*Pros: Reliability and supports 6LoWPAN networks.*

*Cons: No mature protocol and has delay due to protocol conversion.*

#### Use Case

In this proposal [53], the Internet Lab Ajou University deployed an agent application over the 6LoWPAN and a PAN coordinator connected to the gateway with PPP interface. The 6LoWPAN environment composed of a gateway and IEEE802.15.4 devices, containing a PAN coordinator. The devices support Hilow [55] routing protocol. The Device Management agent access to 802.15.4 information base, 6LoWPAN MIB, and IP MIB reduced.

## 5. IoT Network Management Platforms

This section describes the most relevant IoT Network Management platforms. There are open-access and open-source testbeds of IoT platforms that accelerate the deployment of IoT technologies (e.g., IoT-Lab [56] and FIT IoT-LAB [57]). These approaches are used to simulated large number of devices in an IoT environment, but the data obtained does not mirror the real scenario due to network latency. Thus, these category of IoT Network Management platforms was not considered in the scope of this survey because it is impossible to evaluated in the considered real environment.

### 5.1. IMPReSS

The IMPReSS project is a partnership between the European Union and Brazil (EU-Brazil).

The goal of the project was to provide a development platform that allows low-cost development of IoT complex systems and facilitates interaction with users and external systems [45].

The IMPReSS project ended on 31 March 2016.

The IMPReSS development platform can be used by any system that adopts the Smart Society context. The demonstration and validation of the IMPReSS platform will be carried out on energy efficiency systems to reduce the use of energy and CO${}_{2}$ emission in public buildings. One contribution will be the inclusion of intelligence in monitor and control systems, as well as the stimulation of user awareness in reducing energy expenditures. For the configuration management, the PIC-A exposes two ad-hoc commands, in Table 2. The first command provides a list of management data, in XML format, associated with every variable, i.e., the type, the current value, and a list of values to assign. The second command updates values associated with a variable when this is writable [58].

The application interacts with PIC-M that provides setConfiguration and getConfiguration commands to write and read the configuration in any PIC-A. When setting information in parameters, the setConfiguration should be called, passing the XML used to insert new configuration values.

*Pros: Supports the IoT-PIC protocol.*

*Cons: No basic security and no support of the commercial protocols.*

### 5.2. OpenNMS

OpenNMS (Network Management System) open-source platform [34] is used to the management and monitoring of business networks. Developed under the FCAPS (Fault, Configuration, Accounting, Performance, Security) network management model, it is distributed under the GPL license.

OpenNMS is written in Java, in addition to using database PostgreSQL or RRDTool, specifically JRobin (Java port for RRDTool), and supports Red Hat, Debian, Fedora, Mandriva, SuSE, Solaris, Mac OS X and Microsoft Windows.

**Figure 13:**
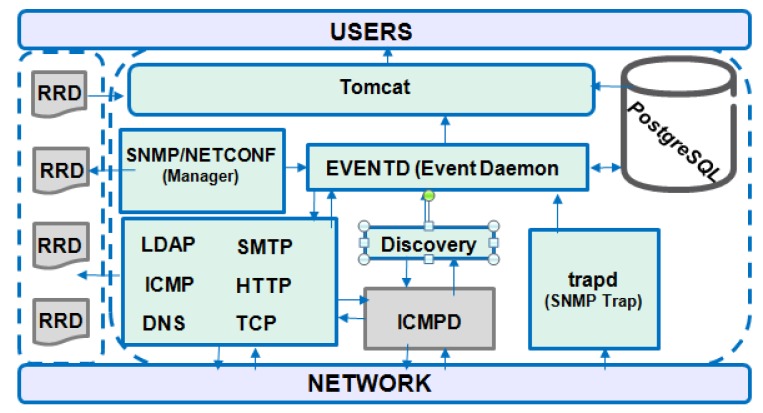
Illustration of the OpenNMS architecture.

The architecture presented in Figure 13 has the features to determine the availability and latency of services, storage and collecting of data, event management (such as SNMP traps), alarms and notifications.

It uses two flows for data collection in Round Robin Database (RRD). The first is through so-called monitors that connect to a network resource and perform a test to verify if it responds correctly. If this does not happen an event is generated. The second flow is through the use of so-called collectors, which can be collected by SNMP (native protocol), NETCONF, Java Management Extensions (JMX), and HTTP.

The generated events are of two types; those generated internally by OpenNMS and those generated externally by SNMP traps, which are characterized according to their description and gravity [59].

*Pros: It supports many Operation Systems and open source.*

*Cons: No basic security and support SNMP native.*

### 5.3. OpenDayLight

The OpenDayLight (ODL) is an open-source Web-platform for network management as SDN. It uses open protocols to allows centralized control and network device monitoring [41]. The ODL supports OpenFlow and offers ready-to-install modular network solutions. There is support for a wide range of network protocols, including SNMP, NETCONF, RESTCONF, OVSDB, Border Gateway Protocol (BGP), Path Computation Element Protocol (PCEP), Locator ID Separation Protocol (LISP), and more. OpenDaylight is slightly different from other controllers because it offers other protocols such as southbound interfaces, e.g., OpenFlow, BGP, and PCEP. In addition, OpenDayLight offers interfaces with OpenStack and Open vSwitch (OVSDB).

**Figure 14:**
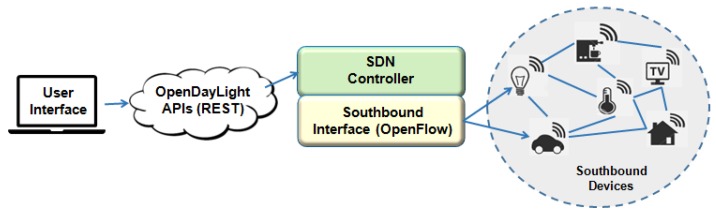
Illustration of the OpenDayLight architecture.

OpenDaylight is a micro-service that uses the sharing of YANG-based (NETCONF) data structures for messages exchange and data storage, as shown in Figure 14. According to Haleplidis et al. [60], through a model addressed to the Model Driven Service Abstraction Layer (MD-SAL), can aggregate any application or function to a service and loaded by the controller.

*Pros: It supports the interoperability of the networks and many native protocols.*

*Cons: No basic security.*

### 5.4. Zabbix

**Figure 15:**
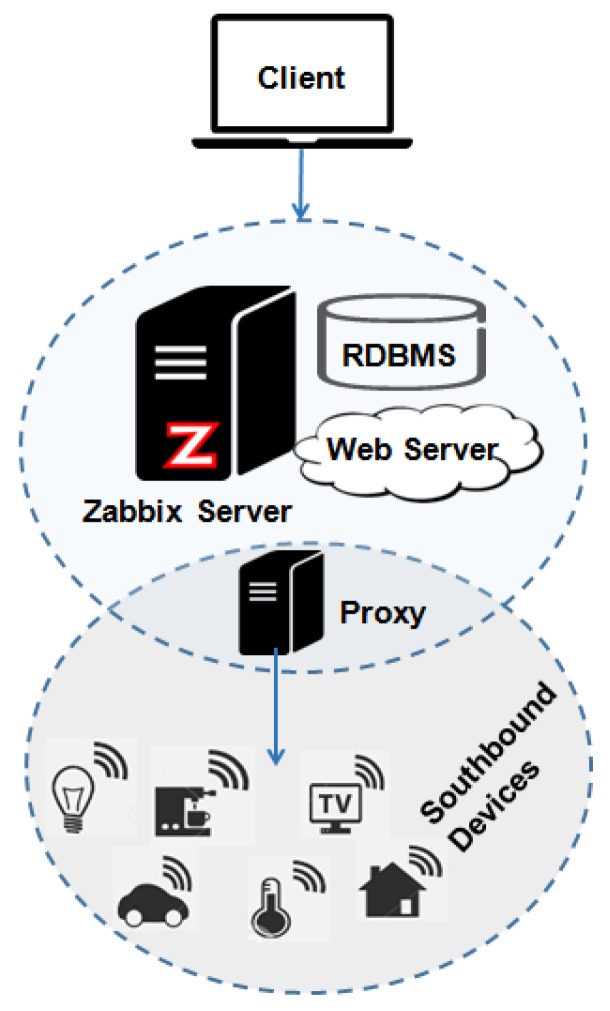
Illustration of the Zabbix architecture.

Zabbix is an open-source tool distributed under the GNU GPLv2 license for network management. It monitors the network services status as well as servers or other hardware. As described in [61], it is characterized as being a centralized management system with semi-distributed monitoring. In Figure 15, the organization can be divided into three main modules.

The platform architecture is distributed and consists of a central server in charge of administering the system and dealing with the interaction between the other two main components: (i) the “Zabbix Agent” to monitor local resources and applications and send them to the server, and (ii) the “Zabbix Proxy” is an optional part of the Zabbix configuration essential for distributed monitoring [62]. Zabbix proxy collects the data from the hosts and stores them in a database of their own to avoid loss of information if there is a problem with the communication with the server. The alert system includes three channels for sending notifications via email, SMS, and jabber (currently called XMPP—Extensible Messaging and Presence Protocol).

*Pros: Mature platform and has a greater number of management metrics.*

*Cons: No basic security and only monitoring the networks.*

## 6. IoT Device Management

Device management has two main components: (i) Device Manager and (ii) Device Agent. The Device Manager is a system that communicates with devices through multiple management protocols and provides individual and bulk device controls. It also manages the device to block remotely when necessary [63].

According to Zehao Liu et al. [64], the Device Agent is a generic component suite that provides management of devices and utilities such as: (i) communication adapters for HTTP and MQTT; (ii) registration of devices; (iii) token management, and (iv) type of management platform.

The managed devices need to maintain and map the device’s identity to their owners. Thus, it allows management through installed software, enabling/disabling functions, monitoring the device availability, and control the security features. Other functions should show be the location and, if available, locking the device remotely, among others. Unmanaged devices have not any management agent and can communicate with the network. Semi-managed devices implement some parts of the managed devices, e.g., only feature control, but not software management [65].

## 7. IoT Device Management Protocols

### 7.1. COnstrained Networks and Devices MANagement (COMAN)

The COMAN Group from the IETF [66] proposes Mobile Object (MO) solutions that simplified MIB, SNMP-based on messages, and CoAP-based management, which could be the protocol used for management of constrained networks and devices.

In Table 3, some Device Management candidate technologies were identified and described:

This survey limits the study to CoAP, OMA-LwM2M, and OMA-DM, but there are several candidates for COMAN technologies.

#### 7.1.1. CoAP—COnstrained Networks and Devices MAnagement

CoAP is an easy to use protocol intended for devices with constrained resources and in conformation with the REST Style. It is a specialized Web transfer protocol designed for M2M applications. It was developed to be used along with lower-level protocols and has been used in many IoT candidates along with IPv6 and UDP.

Also, this protocol meets most requirements for COMAN, such as group-based provisioning, capability discovery, support for energy optimized protocols, unreachable devices and lossy links [66,67].

**Figure 16:**
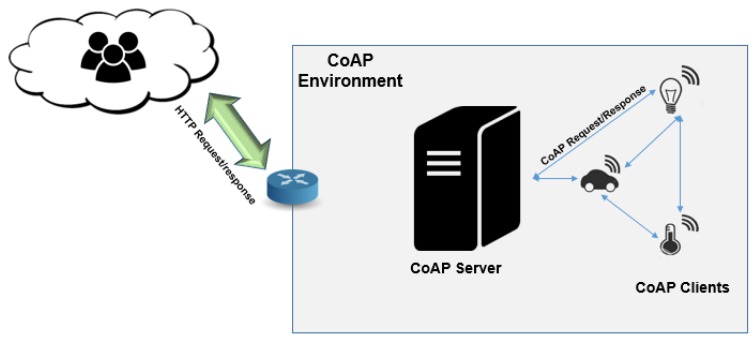
Illustration of the CoAP architecture.

As shown in Figure 16, the CoAP architecture abstracts all network elements as resources, called Universal Resource Identifier (URI) [68]. Inside CoAP management features, it can detect, with low complexity, if a device is online with a simple CoAP ping and verify if the server is stateless.

Also, in the fog computing architecture [7], it is possible to see the performance of this protocol compared to NETCONF and SNMP. This protocol can be used along DTLS (Data- gram Transport Layer Security) [69].

*Pros: Standard communication model and secure.*

*Cons: No supports the heterogeneity networks.*

#### 7.1.2. OMA-DM—Open Mobile Alliance Device Management

OMA-DM provides the management information for connecting devices with the DM tree model [70,71,72] and remotely managing connected devices through the OMA-DM management protocol [73]. It provides efficient methods to manage connected “things” in network environments using: (i) configuration maintenance and management, (ii) configuration of user preferences, (iii) fault detection, query and reporting, (iv) non-application software download, (v) provisioning, and (vi) software management.

**Figure 17:**
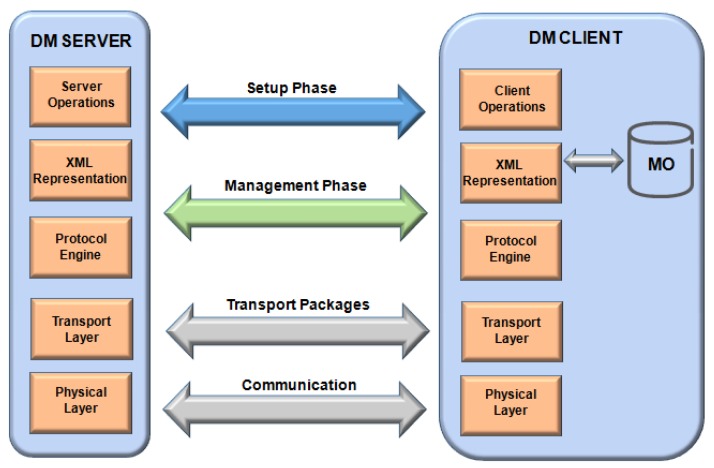
OMA-DM standard management architecture.

The OMA Device Management is divided into DM Server and DM Client devices [74]. Th standard format for communication messages and data transports uses the XML format for the following technologies: physical layers lines or wireless networks (GSM, IrDA, Ethernet or Bluetooth) and transport layers over Wireless Session Protocol (WSP)/WAP [75], HTTP [76], OBEX [77] or similar transports, as shown in Figure 17.

OMA-DM performs data exchange and Device Management with XML data through a DM server/client communication [78]. The OMA-DM consists of two phases: (i) a configuration phase, after authentication enables the exchange of device information through the user commands (Add, Alert, Copy, Get, and others) sent to the DM Client; (ii) the management performs the request/response messages (Status, Generic Alert, and Results) between DM server/client.

*Pros: Standard communication model.*

*Cons: No supports the heterogeneity networks and security.*

#### 7.1.3. OMA-LwM2M—Open Mobile Alliance for Lightweight M2M

**Figure 18:**
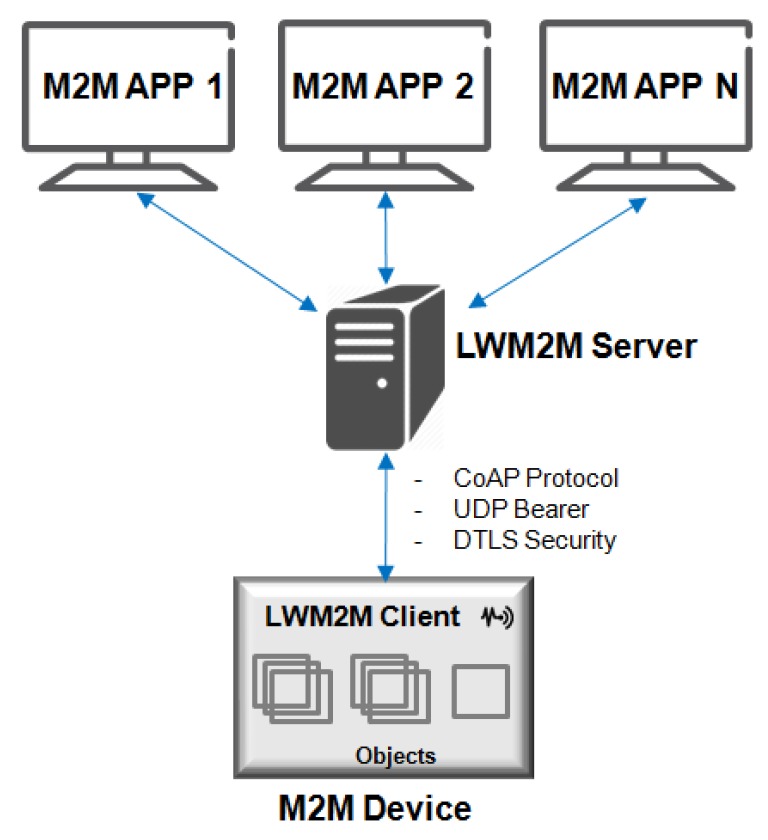
OMA LWM2M standard management architecture.

The OMA LWM2M enables M2M Device Management, acting as an OMA-DM successor using the same protocol, and provides a compact and secure communication for this management [79]. It provides a sub-layer to allow management of LWM2M Server/Client, using a CoAP client-server architecture over UDP as a transport layer, as shown in Figure 18.

The M2M Service Provider, Network Service Provider, or Application Service Provider can be hosted by the LWM2M Server that provides a private or public data center [80]. The LWM2M Client is integrated into a software or device [81]. The LWM2M communication model [82] uses the CoAP methods (GET, PUT, POST, and DELETE) with bindings over UDP transport layer.

*Pros: Communication model and secure.*

*Cons: No supports the heterogeneity networks.*

#### Use Case

Nowadays, there are several solutions (CoAP, OMA-LwM2M, or OMA-DM) with COMAN requirements, e.g., energy states, logging, system authentication, peripheral management, and access controls to the system [66]. Sprint is globally one of the best examples of a mobile Operator that has made FOTA part of its services strategy.

It is fully committed to providing FOTA updates according to the OMA-DM standards [83].

### 7.2. Things Management Protocol (TMP)

The TMP uses the operations get/set, similar to the SNMP operations, to enable default interface for communication between the “*things with things*” and “*things with the applications*” [84].

**Figure 19:**
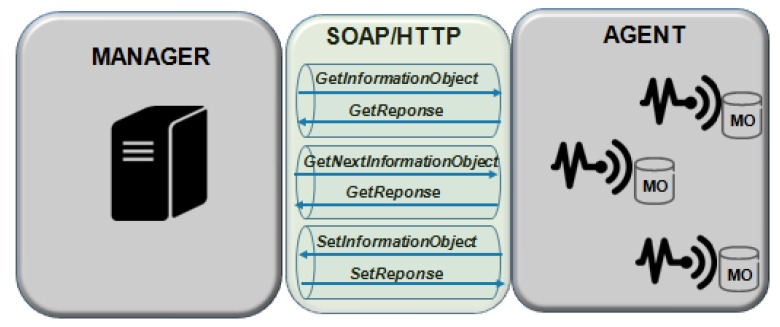
Illustration of the Things Management Protocol architecture.

Guiping et al. [85] describes that the motivation for creating TMP was the need to manage the heterogeneity devices independently. TMP is SOAP-based, as shown in Figure 19, and uses key technologies such as HTTP, XML, and SOA for information integration and connection application based on independent protocols.

In Table 4, the TMP creates the connection between protocol and transport layer protocols and includes several operation request/response messages in the protocol, e.g., GetInformationObject and SetInformationObject.

TMP supports three operations: GetInformationObject, GetNextInformationObject, and SetInformationObject. The basic requirements for operating “things information” are satisfied in these operations.

*Pros: Simple connection and request information.*

*Cons: No robust and secure.*

#### Use Case

The Smart Street Lighting System [86] can be managed remotely using Thing Management Protocol and some tasks can be automated with the objective of reduction of the power consumption, which has an ecological implication.

### 7.3. CPE WAN Management Protocol (CWMP)

**Figure 20:**
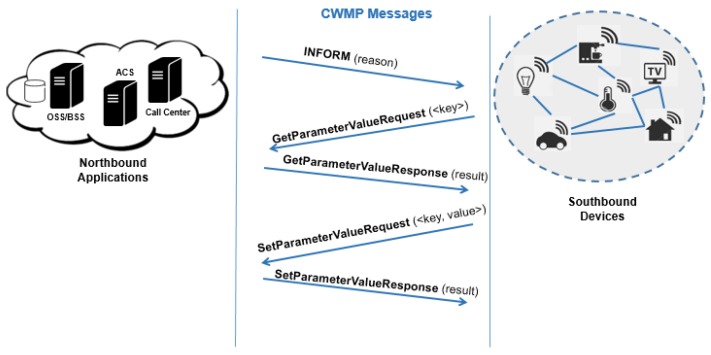
Illustration of the CWMP Protocol messages.

Technical Report 069 (TR-069) is a specification that defines an application layer protocol for Device Management. It was initially published by Broadband Home Working Group and received the name of CPE WAN Management Protocol (CWMP). CWMP is an IP-based protocol and uses XML for all messages, as presented in Figure 20. It provides transaction confidentiality over Transport Control Protocol (TCP) with Secure Sockets Layer (SSL) or Transport Layer Security (TLS) and allows levels of authentication. The protocol uses Hypertext Transfer Protocol (HTTP) and Simple Object Access Protocol (SOAP) based on Web services. The data models standardized and security methods are advantages of CWMP over SNMP.

This protocol works between CPE (Customer-premises equipment) and the Auto-Configuration Server (ACS), achieving better scalability and cost reduction results. Many CPEs can be managed simultaneously by ACS because the session starts and short times are reserved for CPE [87]. The security of this protocol depends on ACS [88,89]. A problem with this protocol is the scalability of the high volume of CPE for a single ACS. Thus, there is a proposal of addiction the components of the ACS management architecture using dynamic grouping, and sub-ACS structure [90].

The ACS can control the CPE through the *get* and *set* methods as parameter values. In the first message, the CPE sends CPE information, e.g., identification, manufacturer, and serial number to the ACS. Then, ACS sends a request with parameters for CPE to execute. After receiving all the answers or does not have requests, CPE closes the session. An Inform message initialize the management session. The client identifies this message, which is confirmed by an InformResponse message by the server. Subsequently, the client can request or assign one or more parameters with a GetParameterValue and SetParameterValue message. Both messages are committed with a SetParameterResponse or GetParameterResponse and the parameter values are updated. Finally, a management session is finalized.

*Pros: Secure auto-configuration and standardize management of devices.*

*Cons: lack of standard for different devices.*

#### Use Case

Incognito ACS is an integrated system to the SAC [91]. It allows to manage copyright of the subscribers, e.g., group of channels or videos on demand over IP. The SAC authorizes TR-069 gateway activation and diagnostics. In the gateway it is possible to execute commands to learn about devices, services, or customer quality. As another use case example, the COSMOS (CPE Operation Support Management and Optimization System) is a CWMP-based Operations Support System (OSS) used to provide integrated multi-function which has an easy to use operating environment. Multi-vendor CPEs (common gateways) are managed by COSMOS, and this system is described in [92].

## 8. IoT Device Management Platforms

### 8.1. Management for the IoT (ManIoT)

**Figure 21:**
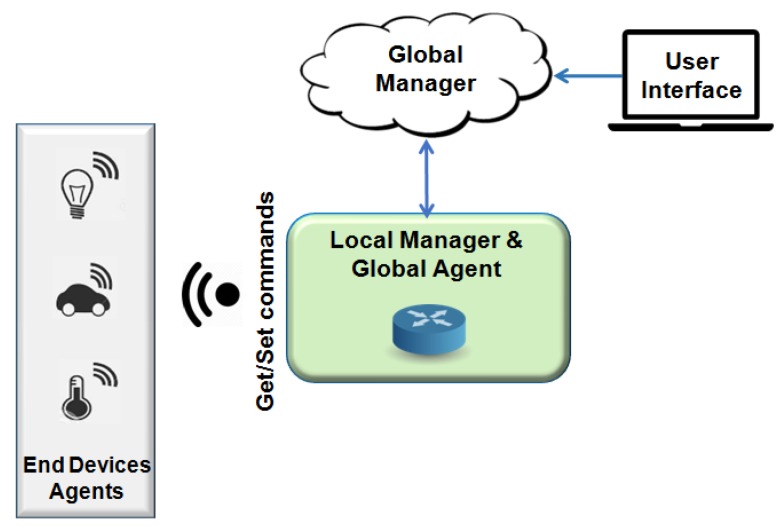
Illustration of the Management for the IoT.

ManIoT platform allows managing devices that make up the IoT environments [93]. Figure 21 shows the applications and sensors installed physically on IoT management environment.

The ManIoT platform takes into account the devices heterogeneity or “things”. Therefore, ManIoT does not require modifications or installations of additional software on devices or applications in user devices. ManIoT accesses the applications through a Web user interface.

The ManIoT standardizes the data model and format used to applications, services, and devices communications. The device’s status (on/off) and the Id (identification device) are characteristics used to model information. To integration with external systems, the platform uses popular protocols and data models of the industry, e.g., XML and RESTful API.

The ManIoT project has two management scopes, Local and Global/Remote. The Local Manager acts to control events performed by a user or application devices that make up a particular scenario, for example, turning a water valve on or off. The remote manager standardizes the actions by users in different scenarios, consumption rates in various areas defined by the water utility.

#### 8.1.1. Local Management

The Local Manager acts based on information on the context within a scenario, i.e., the Local Manager can control and monitor the events, such as turning a lamp on or off.

**Figure 22:**
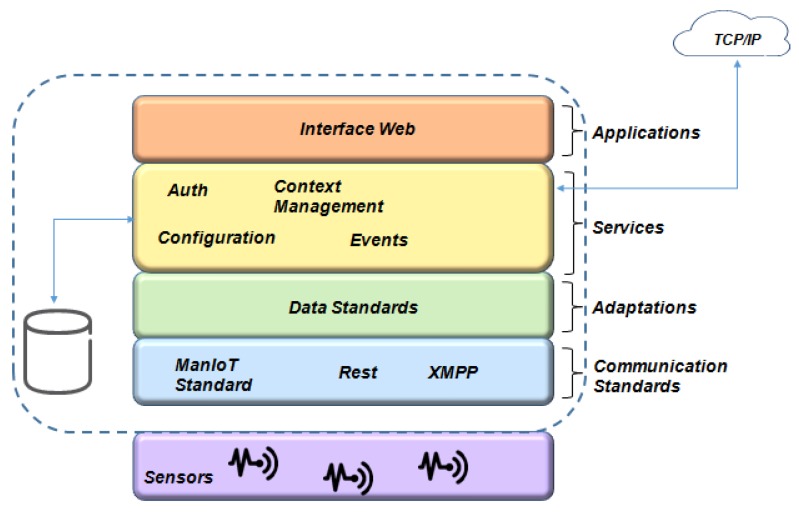
ManIoT: Local Management Architecture.

The functions performed for each layer, as shown in Figure 22, are described below:

- **Application Layer**: The first layer consists of applications that use data provided by one or more devices, as well as platform services. Network users access applications through a Web interface, and these applications, in turn, interact with ManIoT using function calls. Each application requests the platform to perform actions on the sensors based on the implemented scenario, e.g., an energy management application requests turning an air conditioner on or off to reduce consumption.
- **Service Layer**: The second layer is formed by the services that support the applications and use the abstractions implemented by the drivers to communicate with the devices. Among the items in this layer are Storage, Scheduling, Authentication, Settings, Communication, Events, Conflict Management, and Context Management.
- **Adaptation Layer**: This layer is divided into two parts, the first one being responsible for standardizing the data and the second for dealing with the specificities of each device. Each device type has a specific driver that abstracts the specificities of access to its sensors and actuators, which allows the management of the services in an integrated way.
- **Communication Layer**: The layer consists of the different device access protocols. As mentioned earlier, the network may consist of devices that can use different application protocols (i.e., UPnP or proprietary protocol) and different networks (ZigBee, Wi-Fi).
- **A Layer of Things/Devices**: The last layer has the “Things”. There are two devices type: the real devices and the virtual devices. The actual devices are sensors and physical actuators, e.g., an intelligent lamp (actuator), a pressure sensor (sensor). Virtual devices have already captured information from a server connected to a TCP/IP network, e.g., a calendar or email service, or a social networking server.

#### 8.1.2. Global Management

**Figure 23:**
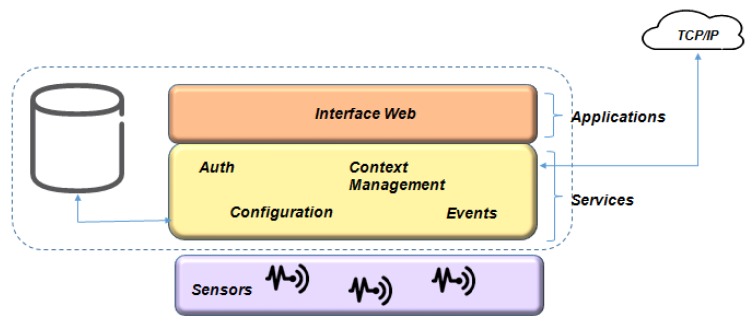
ManIoT: Global/Remote Management Architecture.

The global manager seeks to standardize the actions performed in different scenarios. It has two layers: Application and Services layers, as shown in Figure 23.

The global services have the functions as those development in the local scope, as shown in the second layer of Figure 22 and Figure 23. Global scope services handle larger data sets and provide support for more comprehensive applications. For example, in the context of electrical management, the global manager must have the ability to manage possible power outages in several residences in a neighborhood. The actions defined by the global services are sent and executed in the devices of the respective local managers, using a TCP/IP connection.

*Pros: Context aware and scalability*

*Cons: Privacy, security, supports few native protocols.*

#### Use Case

In the Intelligent Lighting Scenario, the lighting of an environment is adjusted with the presence of people and the existence of natural light. Bulbs are switched conform a person move in the room. Light intensity is inversely proportional to the amount of natural light.

The ManIoT prototype consumes approximately 0.05% of the bandwidth of these networks in the worst case [93]. These values are justified because of the small amount of data exchanged between the prototype of the Local Manager and the devices, thus reinforcing the minimal use of hardware resources.

There were results obtained with scenarios of intelligent lighting and automation of tasks using appropriate metrics to show the capacity of ManIoT to provide a dynamic adaptation and context science to the environment.

### 8.2. Fiware

**Figure 24:**
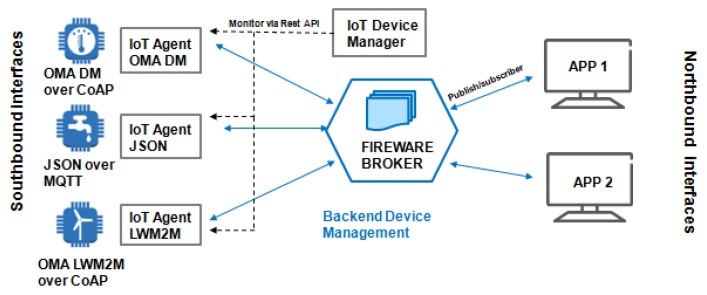
Illustration of the Fiware architecture.

Fiware is an open cloud platform, illustrated in Figure 24, under development and created in a European FP-7 Project to support future Internet. Considered important to several areas, the Fiware has a set of generic enablers (GEs) [94]. According to standard IoT-RA (Figure 1), only the member function is implemented in Fiware Technologies. The platform offers support to various management protocols and standards. It supports OMA NGSI9/10, OMA LWM2M, MQTT, CoAP, and IPv6 [95]. The heterogeneous wireless networks have specific communication protocols to connected devices. Different data encodings make it difficult to find a global deployment.

The platform, illustrated in Figure 24, has a modular architecture that supports several IoT protocols, in which modules are called IoT Agents. However, the integrators must determine the protocol that will be used to connect and select the IoT Agent correct. The IoT Manager collects or sends data to devices that use heterogeneous protocols and translates them to a standard platform, simplifying the Device Management and integration [96].

*Pros: Simplicity and interoperability.*

*Cons: Vulnerability, privacy, and security.*

#### Use Case

The Fiware project is used for orthopedics, podiatry, physiotherapists, and related health services producing prosthesis. This work is time-consuming, cost-inefficient and causes many inconveniences to patients. The Ortholab aim is to produce advanced scan and manufacturing solutions to the insole sectors. With the Ortholab solution, orthopedists or physiotherapists will be able to take digital information of the patient’s body part in an easy way and specify the parameters to 3D printers [97].

### 8.3. ONEM2M

ONEM2M was first released in 2015 and is a partnership project created to establish access-independent M2M service layers specifications. For the management protocol, it has its own technology called Device Management (DM) and it is also evaluating the possibility to implement OMA-DM, OMA-LWM2M or even CWMP [98,99]. This platform has its own system and protocols, as described in [100].

**Figure 25:**
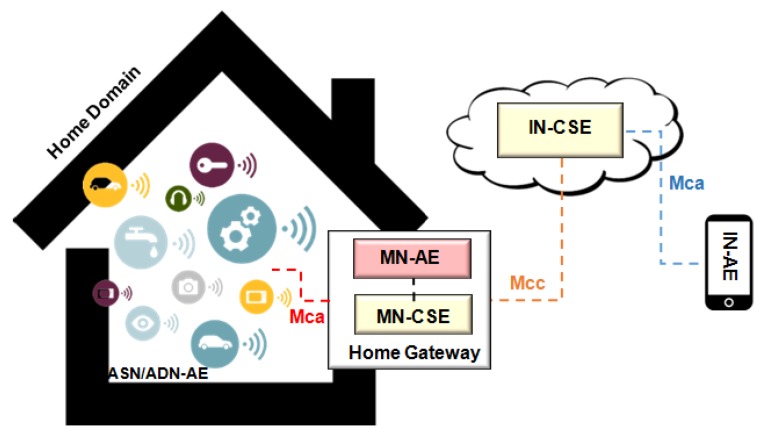
Illustration of the ONEM2M architecture.

Two basic types of entities make up the functional architecture of ONEM2M: AE (Application Entity) and CSE (Common Services Entity), as shown in Figure 25. Northbound and southbound connected devices are considered an AE. The AE needs to be aware of management data protocols or models. Device Management (DMG) enables Device Management capabilities in MNs (for example, M2M Gateways), ASNs, and ADNs (for example, M2M devices). Connected devices residing in an M2M network are managed by services provided by DMG. The information obtained from the AE is used for network administrative actions (e.g., diagnostics, troubleshooting) [101].

The Management Server/Client interface is the Mcc, which uses a Device Management technology (e.g., CWMP, OMA-DM, and LWM2M). Device management technology is used to manage the entities (MN, ASN or ADN, and DMG) and translates requests from other CSEs or from AEs to the Device Management technology. The Mcc interface is technology dependent, as above-described.

*Pros: Interoperability and compliance of services.*

*Cons: Maturity and artificial intelligence.*

#### Use Case

The home lighting use case [102] performs remote control of the lights in a home through a user’s smartphone in the following manner: (i) the lights are deployed and communicate with home gateway; (ii) the home gateway communicates with the cloud platform, making it possible to control the lights remotely with the smartphone; (iii) the cloud platform supports services to enable the smartphone to control the lights, e.g., discovery, data management, group management, publisher/subscriber, and others; (iv) the user’s smartphone hosts an application used to remotely control the lights, i.e., change light state (ON/OFF), discover available lights in the house, among other functions.

### 8.4. SmartThings

**Figure 26:**
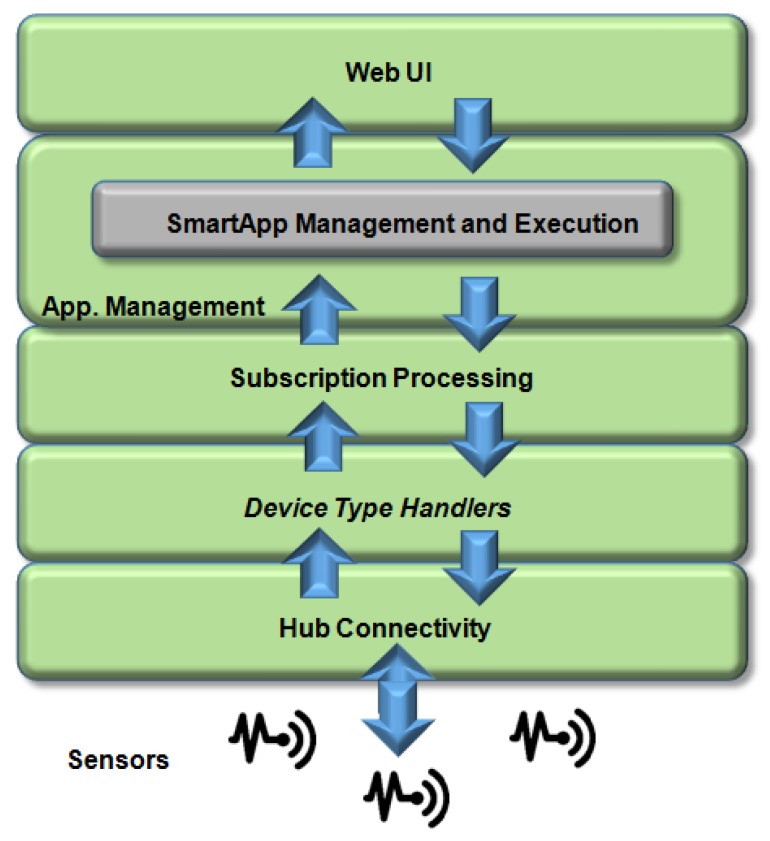
Illustration of the SmartThings architecture.

SmartThings is an open-source solution used to build applications and connect with other devices. It allows new connected applications and supports applications (SmartApps) that communicate with other WebServices through RESTAPI. The SmartThings architecture illustrates the infrastructure blocks that interaction with the devices shown in Figure 26. Communication of devices (sensors and actuators) with application is performed by the HUB entity. The messages are received, identified, and analyzed by a user device on the Device Handler. The response message is discriminated by JSON in SmartThings events. SmartApp handles devices through events managed by Subscription Management.

*Pros: Interoperability and secure services.*

*Cons: It is not an open-source platform and uses a specific API.*

#### Use Case

The SmartThing project is used to optimize simple tasks in daily life. Between the functions used, the following were identified: presence sensors for security and light control, scheduling of house cleaning, and a sensor to get notifications when mail is received [103].

### 8.5. RestThing

The RestThing platform [104] is a Web service infrastructure based on REST with the purpose of hiding the devices heterogeneity and integrate devices into a network. This platform enables developers to build applications accessing physical and Web services, which are both manipulated by a REST-style interface.

**Figure 27:**
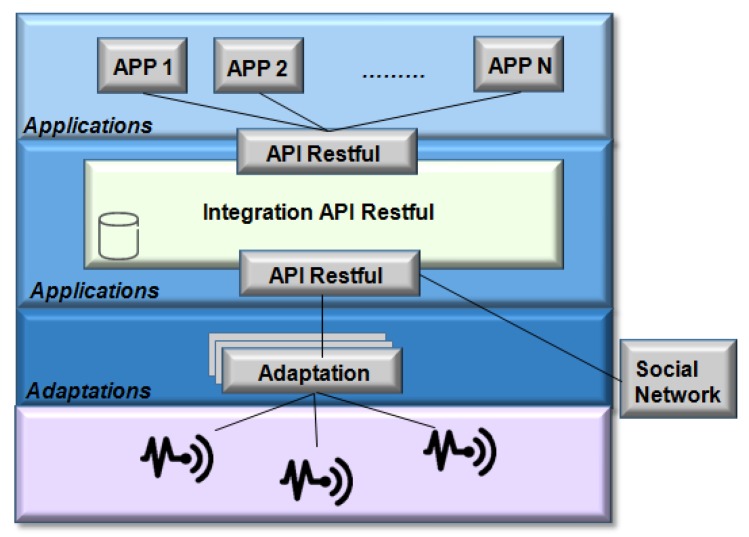
Illustration of the RestThings architecture.

As shown in Figure 27, the RestThing elements are: (i) applications; (ii) RESTful API; (iii) service provider; (iv) adaptation layer; (v) embedded devices, and; (vi) Web resources.

The RESTful API transmitted the data between sensors, gateways and Web applications using three types of data formats: JSON, XML, and CSV. For access to RESTful objects, the HTTP protocol operations used are: the GET method, used to retrieve the current device state; the PUT method, used to modify this device state; the POST method, used to create a new device; DELETE to remove a device, and, in addition, the LIST method, which allows all devices connected to the platform to be listed.

*Pros: It hides device heterogeneity and provides a way to integrate devices into Web applications.*

*Cons: Security and Device Discovery are challenges.*

#### Use Case

The Monitor Temperature and Heartbeat application is the user interface in a smartphone that combines physical and Web resources in the RESTful API. The real-time data view is used to obtain current data from WSNs. The smartphone updates this information by sending a GET to the gateway. The device number of the temperature sensor is what gets current internal lab room temperature as used in Smart Health environments [105].

### 8.6. Xively

Xively provides an API for managing data from the sensors/devices through cloud services. It allows historical data and provides events based on the data generated by sensors/devices. It was created based on the EcoDiF platform [24], Based on REST principles and Web standards such as HTTP and URIs. To minimize the incompatibility among different devices, the platform provides standardized interfaces. The data is organized into data points, streams, and feeds. A feed represents an environment data (i.e., a room) with its data streams, representing data sent by a particular sensor in that environment (i.e., temperatures of the monitored environment).

**Figure 28:**
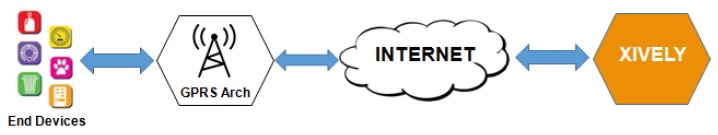
Illustration of the Xively architecture.

Xively is a commercial and closed source solution [106]. There are little details about the architecture of this platform, which is shown in Figure 28. The sensors send data to the platform in JSON, XML or CSV formats using the REST API, via sockets and through the MQTT protocol [107]. However, it is known that there are three ways to manage the devices connected to Xively. In the first case, through the methods implemented by HTTP, the GET method is used by a client program to retrieve data from a feed or data streams. The PUT method is used in the connected devices to send data. In the second case, a socket can be created to avoid the overhead of opening and closing HTTP communications under conditions in which too much data is exchanged. In addition, the first two cases allow the use of the SSL/TLS protocol to provide authentication and data encryption. Finally, the third case uses the publish and subscribe features of the MQTT protocol to send and retrieve data from devices.

*Pros: Integrate with devices easily.*

*Cons: Displays the minimum device notification service.*

#### Use Case

A scalable platform that addresses need residential customers and contractors were developed by SunStat Connect Thermostat, called Watts Water’s SunTouch [108].

The Xively IoT Platform is used to power the remote connectivity of SunStat thermostats, which work with all SunTouch heating products. Furthermore, the Xively provides nearly instantaneous response times from the devices, while not sacrificing stability and reliability that consumers would expect from the heating control device. The Xively platform developed remote connectivity in the SunStat application, enabling consumers to control SunStat devices from a Web browser or mobile device from anywhere in the world connected to the Internet.

Another application is Blueprint [109], that consists of a scalable object directory model with a fast MQTT-based messaging broker. It has a secure provisioning process, which supports millions of secure connections between people, devices, and data around the world.

### 8.7. Carriots

**Figure 29:**
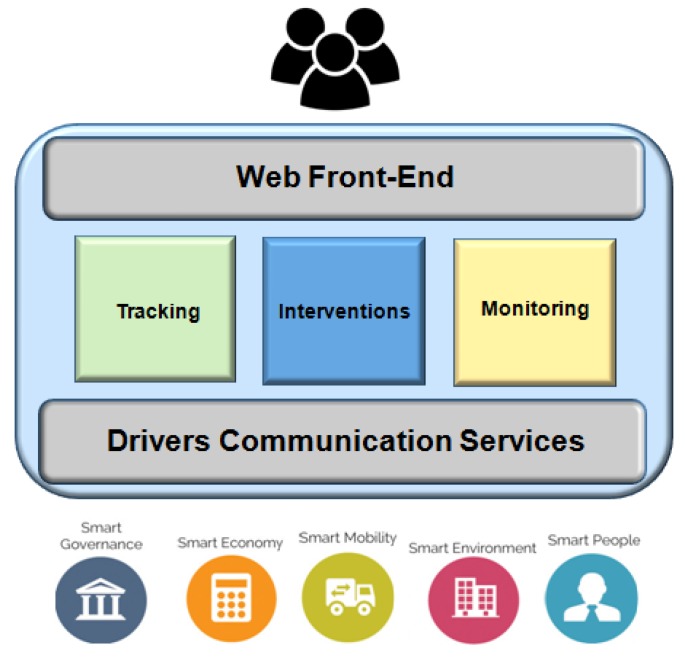
Illustration of the Carriots architecture.

Carriots is an IoT platform that manage data devices provided with cloud services, and that also connects devices to other devices and systems [110]. Therefore, if a system is connected to the platform, it can also be modeled as a device. From its RESTful API, Carriots aim to collect and store any data originated from the most diverse devices. The application engine can guarantee availability to its users no matter the volume of connected devices. These connected devices are associated with services (i.e., physical devices or other resources) and all services belong to a project. As shown in Figure 29, the logical architecture of Carriots consists of the following modules: (i) the REST API; (ii) Big Data; (iii) Project and Device Management; (iv) Business Rules and Event Processing; (v) Security; (vi) Logs and Debug; (vii) Control Panel, and; (viii) External Communication Module.

Data exchanged between devices, connected systems, and the platform can be represented in two different ways: (i) the sensors send data in JSON or XML formats (in a particular platform format) using the REST API, or (ii) through the MQTT message protocol [111].

The Project and Device Management module contains the projects created by users and provides device and its software management, i.e., device provisioning, enabling and disabling devices, and updating firmware. Storing and executing events in the form of scripts created using the Groovy programming language and using if-then-else rules is the responsibility of the Business Rules and Event Processing module.

*Pros: Application used to trigger the other functionality are supported*

*Cons: less friendly user interface.*

#### Use Case

Nowadays, cities have the challenge of improving, protecting the environment, decreasing energy use, and reducing CO${}_{2}$ emissions, as well as having to detect and correct any excesses in light consumption or in water spillage and control expenses.

Carriots City Life is an IoT platform that works like the city brain. It collects data from different sensors or information reported by the citizens and mixes it all to better manage municipal services [112]. it is a cloud platform that allows people to collect, integrate, store and analyze all the city data with a global vision.

## 9. Performance Evaluation, Discussion, and Open Issues

Main characteristics are based on studied management solutions, a qualitative study is performed to characterize management types and technologies used in the most relevant management protocols and platforms. Table 5 and Table 6 summarize the main protocols characteristics considering their standardization resources, data, transport stacks, among others.

The IoT Network Management protocols (Table 5) were originated by the IETF for management of connected devices. The SNMP protocol was the basis for the creation of other protocols such as IoT-PIC and LNMP. Its simplicity in data modeling makes it a fast and a simple configuration protocol. NETCONF was created to be the successor of SNMP using the XML standard for request and response messages. The secure connection transport on SNMP is relevant because its ease of configuration in some scenarios. With the emergence of SDNs, new network management protocols have been proposed, such as OVSDB. It uses JSON technology to expose its devices and network data for the systematic integration of applications.

The IoT Device Management protocols (Table 6) were developed with Internet standards by the IETF. The TLS protocol and SSL protocol are used to secure transport of the information in the network as HTTPS and SSH protocols. In reference to the data modeling and encoding, the OVSDB and COMAN protocols use current Web technologies, such as XML and JSON, that expose network and device information to access other applications through a user name and password.

Table 7 summarizes a comparison among the most relevant IoT Network Management platforms, considering IMPReSS, OpenNMS, OpenDayLight, and Zabbix.

All the platforms have Web user-interfaces and open-source technologies, except IMPReSS (which was finalized in 2016). OpenDaylight supports more IoT Network Management protocols compared to other previously researched solutions. Zabbix is a popular platform for monitoring and management networks and differ in XMPP and NETCONF protocol with OpenNMS.

Table 8 summarizes a comparison among the most relevant IoT Device Management platforms, considering Xively, OneM2M, ManIoT, and other important solutions, regarding several important protocols and types of management approaches.

Despite attending to most requirements, SmartThings believe that supporting widely used protocols and Web technologies is enough to mitigate the problem of heterogeneity devices. However, support for other protocols is an important requirement for Carriots and Xively platforms. Other requirements (like context-awareness and dynamic adaptation) are barely discussed. Context-awareness is an approach to the inclusion of semantic data in a platform, e.g., location and collection time.

Platforms and new solutions are based on the SNMP architecture protocol, as shown in [115,116], such as the use of the OVSDB protocol for the SDN architecture and the XMPP protocol shown in IoT-PIC. SNMP uses more efficient resources thus responding to a processing request up to ten times faster than NETCONF according to the study presented in [7,117,118]. The OpenDayLight modular platform supports more network protocols for IoT, such as SNMP, NETCONF, IoT-PIC/XMPP, OVSDB, and additional features. Then, it can be considered the best platform among the studied IoT Network Management platforms.

**Figure 30:**
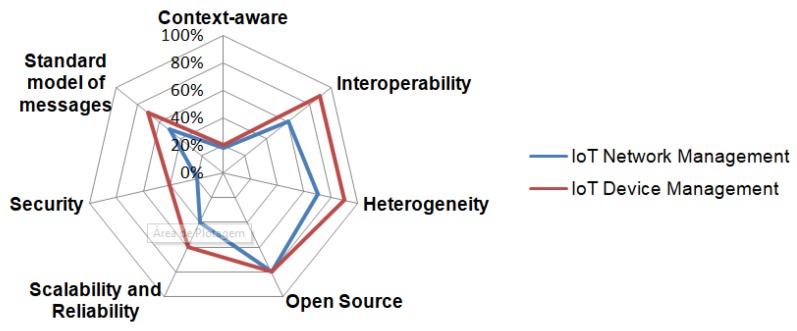
Percentage of resources used in IoT management protocols and platforms.

Figure 30 presents the percentage of the main resources obtained from the surveyed protocols and platforms for IoT management. The studies presented in [6,11,93,119] show the percentage of use of the main raised resources are accounted and demonstrated.

As may be seen, almost all the IoT Device Management platforms solved the heterogeneity and interoperability issue but, for network management, it stills a challenge given they only propose 6LoWPAN network management but SDN and Mesh networks are not supported. Given the protocols and platforms, only about 50% used a standard communication model of networks and devices. Security and context awareness of networks and devices are challenges that stills open. Both types of management have an adoption of open-source protocols and platforms with about 60% of all the surveyed technologies. Some protocols or platforms have a range of features but requirements such as security and interoperability are developed in different ways for each one. Thus, no IoT management platform and protocol meets all the requirements.

Based on this study, it is concluded that IoT Device Management stills in an early stage. The requirements have not been completely explored. Nevertheless, ManIoT and ONEM2M can be considered the best open-source solutions. ONEM2M supports a wide range of management protocols. Xively is the best proprietary platform according to the mapped characteristics. No solution can cover all the requirements of a RAs. Thus, there are open research issues due to divergences between the available technologies and research approaches.

### Open Issues on IoT Management

Considering the previous discussion regarding the available management protocols and platforms for IoT, this section identifies open research issues on IoT resources management. They are presented as follows:

1. Performance evaluation metrics: other performance metrics may be considered or proposed for performance comparison of the available solutions. Thus, a comparative study using error probability, mean response time, and latency may be considered to evaluate the performance of a given solution.
2. Energy saving: extending the lifespan of IoT applications is a matter that may be considered. Connected devices have limited capabilities and should not be overloaded with high throughput. Thus, there is a need to transmit simpler and smaller packets while not overlooking device security.
3. Security: it is a key issue to favor the advancement of IoT. However, due to devices heterogeneity, each company uses different security protocols. Therefore, standardization is an important challenge. Moreover, as the importance and popularity of IoT devices increases and people provide more information on the topic, scammers and the most experienced cyber-criminals continue to search new ways to attack and compromise devices. Consequently, research related to this topic is on the rise in the research community.
4. Real-time management: various types of IoT application domains require high network availability. Health applications, for example, have a critical degree of availability and, therefore, require real-time management. However, limited device resources and power savings are related features for a management solution that should not be overhead in its communication within the network.
5. Interoperability: There are currently several types of devices, protocols, and communication technologies that determine the heterogeneity of IoT networks. These devices must communicate and inter-operate to provide a network service to users. Some research studies assume that new gateways must support several protocols. Furthermore, there is still research on low power device networks as shown in [120].
6. Scalability: The number of devices connected to the network increases exponentially and, thus, the scalability of IoT networks is a critical requirement. The solutions found in literature do not address features regarding scalability. With this, IoT Network Management must support scalability due to the great evolution of IoT devices and related technologies.

## 10. Concluding Remarks

### 10.1. Lessons Learned

This paper analyzed the network and devices management for IoT from different perspectives. The lessons learned from this study are summarized hereafter. First, from the architectural point of view, the IoT Network Management systems is well adopted by IoT RAs and research attempts [6,11]. However, due to the distinct network topologies, heterogeneity between connected devices and protocols without a common standard are possible studies to perform in this context. Scalability and interoperability are priorities in IoT applications and some of the studied technologies present solutions for them [93,121,122,123]. Then, large studies of recent studies were analyzed to discover the main challenges and open issues in IoT. Security and interoperability are top priorities for IoT Network Management systems, followed by performance, reliability, and scalability. Therefore, there are some fundamental features that a network management should provide and they are identified as follows: (i) interoperability between the various devices and platforms available in real environments; (ii) dynamic and adaptation security maintain data integrity to guarantee the availability and QoS during execution; (iii) context-awareness so that information of the locate and state of network objects, is used to perform actions; and (iv) scalability to accept expansion and to operate correctly even in situations of intense use.

Finally, relevant open issues are identified for the purpose of reducing network resources (hardware and bandwidth) as well as infrastructure, security, and energy saving [124,125].

### 10.2. Conclusions

A large number of IoT devices demands management and control solutions for various services. Moreover, the exponential number of connected devices and their inherent constraints motivate the need for efficient management of IoT networks. Therefore, platforms that integrate these services are necessary. However, IoT management current platforms only partially attend to the literature requirements. Overall, this paper presented the concept in detail, its enabling technologies, protocols, platforms, and the recent research addressing IoT management for networks and devices. Among the IoT features, solutions (protocols and platforms) that performs better in terms of scalability, interoperability, security, energy saving, etc. were studied.

Concerning network management, IoT network solutions continue based on the SNMP protocol. The support for each platform search for improving the latency, scalability, and robustness. NETCONF protocol was developed to be the natural successor of SNMP, as SNMP is focused on monitoring and not on network configuration. The OpenDayLight platform can be considered the best solution based on the supported protocols. For IoT devices management, ONEM2M open-source approach and Xively proprietary technology were evaluation with other technologies. It was observed that ONEM2M and Xively predict scalability and promote the integration of devices with local/remote management features always remembering the guarantee the heterogeneity and security.

Finally, the main goal to achieve regarding the best choice for IoT Network Management protocols and platforms for a real IoT management solution was attained since important insights, a detailed description and discussion on the topic was performed. Further research works on the topic were also identified.

## Figures and Tables

**Table 1:** Exchanged messages at the Abstraction layer.

Exchange Messages	Description
ALME-GET	This message is used by the HLEs to get a description of the HLEs device.
ALME-SET	This message is used by the HLEs to send a configuration of the HLEs device.

**Table 2:** Configuration Management Ad-Hoc commands.

Ad-Hoc Commands	Description
getAvailableDrivers	Returns the list of drivers available on the repository.
getConfiguration	This operation is used to get the list of the current
setConfiguration	values in the parameters that can be configured in the component. Updates the component configuration and setting the values passed as a parameter.

**Table 3:** COMAN—Candidate Technologies.

Technology	Description
CoAP	The IETF has defined a binary protocol, the Constrained Application Protocol (CoAP), easy to analyze and especially designed for constrained devices, which is used with lower-level protocols, but it is particularly adapted over UDP/IPv6.
OMA-LwM2M	OMA Lightweight M2M is a Device Management protocol used to M2M networks environment.
OMA-DM	OMA Device Management provides functions for device management. The Device Management happens through communication between a server (Device Manager) and the client (Device Agent) using HTTP transport.

**Table 4:** TMP Operational Requests/Response Messages.

Operational Messages	Description
GetInformationObject	In IoT application, this message is used to read things information object.
GetNextInformationObject	Used to read one or more things information object next to the current object.
SetInformationObject	Used to write one or more things information object. The value of one thing information object is written per one operation.

**Table 5:** Main characteristics comparison of the IoT Network Management protocols.

	SNMP/LNMP	NETCONF	IoT-PIC/XMPP	OVSDB	IEEE 1905
Standard	IETF	IETF	IETF	IETF	IEEE
Resource	OIDs	Paths	URLs	URLs	URLs
Data	SMI	YANG	WSDL	JSON	WSDL
Modeling					
Encoding	BER	XML	XML	JSON	XML
Transport	UDP	SSH/TCP	HTTP,	HTTP,	HTTP,
Stack			Web API	SSL/TLS	Web API

**Table 6:** Main characteristics comparison of the IoT Device Management protocols.

	CoAP	OMA-DM L2M2M	OMA	TMP	CWMP
Standard	IETF	IETF	IETF	IETF	Broadband Forum
Resource	URLs	URLs	URLs	URLs	URLs
Data Modeling	JSON	XML, JSON	XML, JSON	WSDL	XML
					WSDL
Encoding	JSON	XML, JSON	XML, JSON	XML	XML
Transport	UDP	UDP	UDP	HTTP	HTTP
Stack	HTTPS	HTTPS	HTTPS	Web API	SSH/TCP
	SSH/TCP	SSH/TCP	SSH/TCP		

**Table 7:** Main characteristics comparison of the IoT Network Management platforms.

	IMPReSS	OpenNMS	OpenDayLight	Zabbix
SNMP		X	X	X
NETCONF		X	X	
IoT-PIC/XMPP	X		X	X
LNMP		X		X
OVSDB			X	
IEEE 1905				

**Table 8:** Main characteristics comparison of the IoT Device Management platforms.

	YOAPY [113]	EcoDIF [24]	RestThing	SmartThings	IFTTT [114]	ManIoT	Xively	Carriots	Fiware	ONEM2M
OpenSource		X	X	X	X	X			X	X
Heterogeneity		X	X	X	X	X	X	X	X	X
Security and										
Privacy		X		X	X	X	X	X	X	X
Scalability and										
Reliability		X	X	X	X	X	X	X	X	X
RFID	X		X			X	X	X	X	X
CoAP	X	X	X	X	X	X	X		X	X
OMA-DM	X			X	X		X			X
OMA-L2M2M	X			X	X		X			X
TMP										
CWMP							X			X
6LoWPAN	X					X	X	X	X	X
SOA		X	X	X			X	X		X
Data										
Management	X						X	X	X	X
Device										
Management		X	X	X	X	X	X	X	X	X
Local										
Management	X			X		X	X	X	X	X
Global										
Management	X	X	X	X	X	X	X	X	X	X
Remote										
Management	X	X	X	X	X	X	X	X	X	X
Context										
Management					X	X	X	X	X	X

## References

[1] Bonaventure O. (2011). Computer Networking: Principles, Protocols and Practice.

[2] Zanella A., Bui N., Castellani A., Vangelista L., Zorzi M. (2014). Internet of Things for smart cities. IEEE Internet Things J..

[3] Singh K.J., Kapoor D.S. (2017). Create Your Own Internet of Things: A survey of IoT platforms. IEEE Consum. Electron. Mag..

[4] Du X., Xiao Y., Guizani M., Chen H.H. (2007). An effective key management scheme for heterogeneous sensor networks. Ad Hoc Netw..

[5] Ngu A.H., Gutierrez M., Metsis V., Nepal S., Sheng Q.Z. (2017). An effective key management scheme for heterogeneous sensor networks. IEEE Internet Things J..

[6] Qiu T., Chen N., Lib K., Qiaoc D., Fud Z. (2017). Heterogeneous ad hoc networks: Architectures, advances and challenges. Ad Hoc Netw..

[7] Slabicki M., Grochla K. Performance evaluation of CoAP, SNMP and NETCONF protocols in fog computing architecture. Proceedings of the NOMS 2016—2016 IEEE/IFIP Network Operations and Management Symposium.

[8] Tayur V.M., Suchithra R. Internet of Things Architectures: Modeling and Implementation Challenges. Proceedings of the International Conference, “Computational Systems for Health & Sustainability”.

[9] Benamar N., Jara A., Ladid L., Ouadghiri D.E. Challenges of the Internet of Things: IPv6 and Network Management. Proceedings of the Innovative Mobile and Internet Services in Ubiquitous Computing (IMIS).

[10] Atzori L., Iera A., Morabito G. (2010). The Internet of Things: A survey. Comput. Netw..

[11] Meng M., Ping W., Chao-Hsien C. Data Management for Internet of Things: Challenges, Approaches and Opportunities. Proceedings of the IEEE and Internet of Things Conference (iThings/CPSCom 2013).

[12] Passemard A. (2014). The Internet of Things Protocol stack—From sensors to businesss value. Internet of Things (IoT) Talks.

[13] Ruiz L.B., Nogueira M.S.J., José M.S., Loureiro A.A. (2003). MANNA: A management architecture for wireless sensor networks. IEEE Commun. Mag..

[14] Pires P.F., Cavalcante E., Barros T., Delicato F.C., Batista T., Costa B. A platform for integrating physical devices in the Internet of Things. Proceedings of the 12th IEEE International Conference on Embedded and Ubiquitous Computing.

[15] Clemm A. (2007). Network Management Fundamentals.

[16] Athreya A.P., Tague P. Network Self-Organization in the Internet of Things. Proceedings of the 10th Annual IEEE Communications Society Conference Sensor, Mesh and Ad Hoc Communications and Networks (SECON).

[17] Lee S., Levanti K., Kim H.S. (2014). Network monitoring: Present and future. Comput. Netw..

[18] Kurose J.F., Ross K.W. (2010). Computer Networking: A Top-Down Approach Featuring the Internet.

[19] Gabdurahmanov M., Trygg S. (2011). Analysis and Evaluation of Network Management Solutions.

[20] Vermesan O., Friess P. (2014). Internet of Things—From Research and Innovation to Market Deployment.

[21] Sanchez L., McCloghrie K., Saperia J. (2001). RFC3139: Requirements for Configuration Management of IP-Based Networks. https://tools.ietf.org/html/rfc3139.

[22] Peterson L.L., Davie B.S. (2012). Computer Networks: A Systems Approach.

[23] Zhou C., Zhang X. Toward the Internet of Things Application and Management: A Practical Approach. Proceedings of the IEEE 15th International Symposium on World of Wireless, Mobile and Multimedia Networks (WoWMoM).

[24] Delicato F.C., Pires P.F., Batista T. (2013). Middleware Solutions for the Internet of Things.

[25] Perera C., Member S., Zaslavsky A., Christen P. (2013). Context Aware Computing for The Internet of Things: A Survey. IEEE Commun. Surv. Tutor..

[26] Song B., Cheong Y., Lee T., Jeong J. Design and Security Analysis of Improved Identity Management Protocol for 5G/IoT Networks. Proceedings of the World Conference on Information Systems and Technologies.

[27] Bauer M., Boussard M., Bui N., Carrez F., Jardak C., Loof J.D., Magerkurth C., Meissner S., Nettsträter A., Olivereau A. Deliverable D1.5—Final Architectural Reference Model for the IoT v3.0. http://www.meet-iot.eu/deliverables-IOTA/D_15.pdf.

[28] Ma Y., Chen J., Huang Y., Lee M. (2010). An Efficient Management System for Wireless Sensor Networks. Sensors.

[29] Case J., Fedor M., Schoffstall M., Davin J. (1990). RFC 1157—Simple Network Management Protocol (SNMP). https://tools.ietf.org/html/rfc1157/.

[30] SNMP Research Simple Network Management Protocol. http://www.snmp.com/protocol/.

[31] Specialski E.S. (2002). Management of Computer Networks and Telecommunications. Master’s Thesis.

[32] Ericsson A.B. Simple Network Management Protocol 5.2.5 (SNMP)-Erlang. http://erlang.org/doc/apps/snmp/snmp.pdf.

[33] ASN.1 PROJECT Introduction to ASN.1. http://www.itu.int/en/ITU-T/asn1/Pages/introduction.aspx.

[34] OpenNMS—Open Networks Management System. https://www.opennms.org/en.

[35] Enns R., Bjorklund M., Schoenwaelder J., Bierman A. (2011). RFC 6241—Network Configuration Protocol (NETCONF). https://tools.ietf.org/html/rfc6241.

[36] Bjorklund M. (2010). RFC 6020—YANG—A Data Modeling Language for NETCONF. https://tools.ietf.org/html/rfc6020.

[37] Xu H., Wang C., Liu W., Chen H. (2012). NETCONF-based Integrated Management for Internet of Things using RESTful Web Services. Int. J. Future Gener. Commun. Netw..

[38] Chappell C. (2013). White Paper Creating the Programmable Network: The Business Case for NETCONF/YANG in Network Devices.

[39] Pfa B., Davie B. (2013). RFC 7047—The Open vSwitch Database Management Protocol. https://tools.ietf.org/html/rfc7047.

[40] Davie B., Koponen T., Pettit J., Pfa B., Casado M., Gude N., Petty T. (2017). Public Review for A Database Approach to SDN Control Plane Design. ACM SIGCOMM Comput. Commun. Rev..

[41] Beryllium R. OpenDaylight Project. https://wiki.opendaylight.org/view/Documentation.

[42] Pastrone C., Boella M., Spirito M., Tomasi R., Rizzo F. (2008). A Jabber-Based Management Framework for Heterogeneous Sensor Network Applications. ACM SIGCOMM Comput. Commun. Rev..

[43] Scheck M. (2015). Performance tests XMPP. IoT Messaging Protocols.

[44] Stanik A., Kao O. A proposal for REST with XMPP as base protocol for intercloud communication. Proceedings of the 7th International Conference on Information, Intelligence, Systems & Applications (IISA).

[45] IMPReSS Consortium—D3.4 Network Management. http://impressproject.eu/downloads/deliverables/D3.4_Network_Management.pdf.

[46] Yan H. (2014). Smart Devices Collaboration for Energy Saving in Home Networks. Préparée à L’unité de Recherche IRISA (UMR 6074). Ph.D. Thesis.

[47] Yan H., Fontaine F., Yan H., Fontaine F. (2014). An adaptive proxy for compliance of the equipments to IEEE 1905. Patent.

[48] IEEE (2013). IEEE Standard for a Convergent Digital Home Network for Heterogeneous Technologies.

[49] Palm S. Connected home—Focus on Networked Power Save and Management. Proceedings of the ACEEE Intelligent Efficiency Conference: Program.

[50] Neves P.A.C., Rodrigues J.J.P.C. (2010). Internet Protocol over Wireless Sensor Networks, from Myth to Reality. J. Commun..

[51] Sehgal A., Perelman V., Kuryla S., Schönwälder J. (2012). Management of Resource Constrained Devices in the Internet of Things. IEEE Commun. Mag..

[52] Ayala G., Poskal P., Gamess E. SNMP JManager: An Open Source Didactic Application for Teaching and Learning SNMP v1/2c/3 with Support for IPv4 and IPv6. Proceedings of the Seventh LACCEI Latin American and Caribbean Conference for Engineering and Technology (LACCEI’2009).

[53] Mukhtar H., Kang-myo K., Chaudhry S.A., Akbar A.H., Ki-hyung K., Yoo S. LNMP—Management Architecture for IPv6 based low-power Wireless Personal Area Networks (6LoWPAN). Proceedings of the Network Operations and Management Symposium.

[54] Kushalnagar N., Montenegro G., Schumacher C. (2007). RFC 4919—6LoWPAN: Overview, Assumptions, Problem Statement and Goals.

[55] Kim K., Yoo S., Park D., Lee J., Mulligan G. (2007). Hierarchical Routing over 6LoWPAN (HiLow).

[56] Fernandes J., Nati M., Loumis N.S., Nikoletseas S., Raptis T.P., Krco S., Ziegler S. IoT Lab: Towards co-design and IoT solution testing using the crowd. Proceedings of the International Conference on Recent Advances in Internet of Things (RIoT).

[57] Adjih C. FIT IoT-LAB: A large scale open experimental IoT testbed. Proceedings of the IEEE 2nd World Forum Internet Things (WF-IoT).

[58] IMPReSS Consortium—D7.3.2 Final Design and Implementation of the Configuration and Composition Manager. http://www.compose-project.eu/sites/default/files/publications/D7.3.2%20-%20Use%20cases%20implementation%E2%80%93%20Final%20version.pdf.

[59] The OpenNMS Group (2015). OpenNMS—Open Network Management System Wiki. https://wiki.opennms.org/wiki/Severity.

[60] Haleplidis E., Hadi Salim J., Denazis S. (2015). Towards a Network Abstraction Model for SDNs. J. Netw. Syst. Manag..

[61] Olups R. (2010). Zabbix 1.8 Network Monitoring.

[62] Dalle Vacche A., Kewan Lee S. (2013). Mastering Zabbix.

[63] Tormo G.D., Marmol F.G., Perez G.M. (2014). Dynamic and flexible selection of a reputation mechanism for heterogeneous environments. Future Gener. Comput. Syst. J..

[64] Liu Z., Liu F., Lin K. Agent-Based Device Management in RFID Middleware. Proceedings of the 4th International Conference on Wireless Communications, Networking and Mobile Computing (WiCOM’08).

[65] Bera S., Misra S., Roy S.K., Obaidat M.S. (2016). Soft-WSN: Software-Defined WSN Management System for IoT Applications. IEEE Syst. J..

[66] Greevenbosch B., Li K., Van der Stok P. Candidate Technologies for COMAN. http://tools.ietfs.org/html/draft-greevenboschcoman-candidate-tech-03.

[67] Lamaazi H., Benamar N., Jara A., Ladid L., El Ouadghiri D. Internet of thing and networks management: Lnmp, snmp, coman protocols. Proceedings of the First International Workshop on Wireless Networks and Mobile COMmunications (WINCOM 2013).

[68] Sheng Z., Wang H., Yin C., Hu X., Yang S., Leung V.C. (2015). Lightweight management of resource-constrained sensor devices in internet of things. IEEE Internet Things J..

[69] Castro M., Jara A.J., Skarmeta A.F. (2016). Enabling end-to-end coap based communications for the web of things. J. Netw. Comput. Appl..

[70] Open Mobile Alliance (OMA) (2010). OMA Device Management Standardized Objects.

[71] Open Mobile Alliance (OMA) (2012). OMA DM Device Description Framework.

[72] Open Mobile Alliance (OMA) (2012). OMA Device Management Tree and Description.

[73] Open Mobile Alliance (OMA) (2016). OMA Device Management Protocol.

[74] Chu N., Raouf D., Corlay B., Ammari M., Gligoric N., Krco S., Ognjanovic N., Obradovic A. (2013). OMA DM v1.x compliant Lightweight Device Management for Constrained M2M devices. Eur. Trans. Telecommun..

[75] Yadav V., Verma M., Nisha (2015). A Survey Paper on Wireless Access Protocol. Int. J. Comput. Sci. Inf. Technol..

[76] Derhamy H., Eliasson J., Delsing J., Priller P. A survey of commercial frameworks for the Internet of Things. Proceedings of the 2015 IEEE 20th Conference on Emerging Technologies & Factory Automation (ETFA).

[77] Pulipati M., Phani S.K. (2013). Comparison of Various Short Range Wireless Communication Technologies with NFC. Inter. J. Sci. Res..

[78] Kang J., Ju H., Choi M., Won-Ki Hong J., Kim J. (2009). OMA DM-based remote software fault management for mobile devices. Int. J. Netw. Manag..

[79] Klas G., Rodermund F., Shelby Z., Akhouri S., Höller J. (2014). Lightweight M2M: Enabling Device Management and Applications for the Internet of Things.

[80] Ocak M.C. (2014). Implementation of an Internet of Things Device Management Interface. Master’s Thesis.

[81] Putera C.A.L., Lin F.J. Incorporating OMA Lightweight M2M protocol in IoT/M2M standard architecture. Proceedings of the 2015 IEEE 2nd World Forum on Internet of Things (WF-IoT).

[82] Rao S., Chendanda D., Deshpande C., Lakkundi V. Implementing LWM2M in constrained IoT devices. Proceedings of the IEEE ICWiSe 2015.

[83] Red Band Software (2011). FOTA Usage in the United States.

[84] Chowdhury S.N., Kuhikar K.M., Dhawan S. (2015). IoT Architecture: A Survey. Int. J. Ind. Electron. Electr. Eng..

[85] Dai G. Design and implementation on SOAP-based things management protocol for internet of things. Proceedings of the 10th World Congress Intelligent Control and Automation (WCICA).

[86] Wisys Technologies EZLux—Smart Street Lighting. https://wisystech.com/solutions/ezlux-smart-street-lighting-bangalore/.

[87] Hillen B.A.G., Passchier I., Matthijssen E.F., den Hartog F.T.H., Selgert F. Remote management of mobile devices with broadband forum’s TR-069. Proceedings of the Telecommunications Network Strategy and Planning Symposium.

[88] TR-69 CWMP v1.4 Especifications. https://www.broadband-forum.org/technical/download/TR-069.pdf.

[89] Husain S., Prasad A., Kunz A., Papageorgiou A., Song J. (2014). Recent Trends in Standards Related to the Internet of Things and Machine-to-Machine Communications. J. Inf. Commun. Converg. Eng..

[90] Lee K., Chu H., Chu J., Lin Y., Hsiao C., Hou T. (2014). ACS management capacity enhancement mechanism in CWMP. Electron. Lett..

[91] Roll Out New Services with TR-069: A Cable IPTV Use Case. http://www.incognito.com/wp-content/uploads/cable-tr-069-iptv-use-case.

[92] Wang T.H., Chen Y.C., Hsu C.M., Hsu K.S., Young H.C. Auto scaling of containerized ACSs for CPE management. Proceedings of the 2016 18th Asia-Pacific Network Operations and Management Symposium (APNOMS).

[93] Santos M., Castro T.O., Macedo D.F., Horizonte B. ManIoT: A Platform for Management of Internet Devices of Things. Proceedings of the Brazilian Symposium on Computer Networks and Distributed Systems.

[94] Stravoskoufos K., Sotiriadis S., Petrakis E. Iot-a and fiware: bridging the barriers between the cloud and iot systems design and implementation. Proceedings of the 6th International Conference on Cloud Computing and Services Science (CLOSER 2016).

[95] FIWARE IoT Stack. https://www.fiware.org/.

[96] FIWARE Wiki. http://forge.fiware.org/plugins/mediawiki/wiki/fiware/index.php/MainPage.

[97] FIWARE Map. http://map.fiware.org/actors/smes/434.

[98] Chang W.G., Lin F.J. Challenges of incorporating OMA LWM2M gateway in M2M standard architecture. Proceedings of the 2016 IEEE Conference on Standards for Communications and Networking (CSCN).

[99] Datta S.K., Bonnet C. A lightweight framework for efficient M2M device management in oneM2M architecture. Proceedings of the 2015 International Conference on Recent Advances in Internet of Things (RIoT).

[100] Swetina J., Lu G., Jacobs P., Ennesser F., Song J. (2014). Toward a standardized common M2M service layer platform: Introduction to oneM2M. IEEE Wirel. Commun..

[101] (2016). Application Developer Guide—TR-0025 V1.0.0. ftp://ftp.onem2m.org/Work%20Programme/WI-0042/TR-0025-ApplicationDeveloperGuide-V202.DOC.

[102] (2017). Application Developer Guide—Use Case. http://onem2m.org/application-developer-guide/use-case.

[103] Use Case for SmartThings Project. SmartThings Prokect. https://blog.smartthings.com/tag/smartthings-use-cases/.

[104] Qin W., Li Q., Sun L., Zhu H., Liu Y. Restthing: A restful web service infrastructure for mash-up physical and web resources. Proceedings of the IFIP 9th International Conference Embedded and Ubiquitous Computing (EUC).

[105] Lavanya S. (2016). A Smart Network: IoT to Monitor Temperature and Heart beat of a Person Using RFID Technology. Int. J. Chem. Sci..

[106] Ray P.P. (2017). A survey of IoT cloud platforms. Future Comput. Inform. J..

[107] Jang R., Soh W., Jung S. Design and Implementation of Data-Report Service for IoT Data Analysis. Proceedings of the International Conference on Chemical, Material and Food Engineering (CMFE-2015).

[108] Wendel A. (2016). Customer Spotlight: Watts Water. Xively Blog.

[109] Lorenz S. (2015). Blueprint: A Central Control Hub for Connected Products. Xively Blog.

[110] carriots.com Carriots–IoT Application Platform. https://www.carriots.com.

[111] Zdravkovic M., Trajanovic M., Sarraipa J., Jardim-Gonçalves R., Lezoche M. Survey of Internet-of-Things platforms. Proceedings of the 6th International Conference on Information Society and Techology, ICIST 2016.

[112] Gluhak A., Vermesan O., Bahr R., Clari F., MacchiaMaria T., Delgado T., Hoeer A., Bösenberg F., Senigalliesi M., Barchetti V. D03.01 Report on IoT Platforms Activities. ICT-30-2015: Internet of Things and Platforms for Connected Smart Objects. http://www.internet-of-things-research.eu/pdf/D0301WP03H2020UNIFY-IoTFinal.pdf.

[113] Jara J.A., Zamora M.A., Skarmeta A.F. Knowledge acquisition and management architecture for mobile and personal health environments based on the Internet of things. Proceedings of the IEEE 11th International Conference on Trust, Security and Privacy in Computing and Communications (TrustCom).

[114] IFTTT—Connect the APPs You Love. https://ifttt.com/.

[115] Neha, Meena M.S. (2016). Rajbir Implementation of SNMP (Simple Network Management Protocol) on Sensor Network. Int. J. Adv. Res. Comput. Eng. Technol..

[116] Huang H.P., Xiao S.D., Meng X.Y. (2015). Applying SNMP Technology to Manage the Sensors in Internet of Things. Open Cybern. Syst. J..

[117] Hedstrom B., Watwe A., Sakthidharan S. (2011). Protocol Efficiencies of NETCONF versus SNMP for Configuration Management Functions. Ph.D. Thesis.

[118] Marotta M.A., Both C.B., Rochol J., Granville L.Z., Tarouco L.M.R. Evaluating Management Architectures for Internet of Things Devices. Proceedings of the 2014 IFIP IEEE—Wireless Days (WD).

[119] Razzaque M.A., Milojevic-Jevric M., Palade A., Clarke S. (2016). Middleware for Internet of Things: A Survey. IEEE Internet Things J..

[120] Silva J.C., Andery F., Mazzer D., Mendes L.D.P. Factorial Design Analysis Applied to the Performance of Transmission Power Optimization Techniques for Wireless Sensor Networks. Proceedings of the XXII Iberchip Workshop.

[121] Bosch Software Innovations (2017). IoT Platforms for Device Management: Positioning of Bosch Software Innovations. Digit. CX IoT I Eur..

[122] Stusek M., Masek P., Zeman K., Kovac D., Cika P., Pokorny J., Kröpfl F. (2016). A Novel Application of CWMP: An Operator-grade Management Platform for IoT. Int. J. Adv. Telecommun. Electrotech. Signals Syst..

[123] (2015). Developer Documentation: Release 1.0. SmartThing Project. https://media.readthedocs.org/pdf/smartthings/latest/smartthings.pdf.

[124] Yaqoob I., Ahmed E., Hashem I.A.T., Ahmed A.I.A., Gani A., Imran M., Guizani M., IEEE Communications Society (2017). Internet of Things Architecture: Recent Advances, Taxonomy, Requirements, and Open Challenges. IEEE Wirel. Commun..

[125] Sinha N., Pujitha K.E., Alex J.S.R. Xively based sensing and monitoring system for IoT. Proceedings of the 2015 International Conference on Computer Communication and Informatics (ICCCI).

